# Unintended Consequences of COVID-19 Public Health and Social Measures in Camps and Camp-Like Settings: A Systematic Review and Conceptual Analysis

**DOI:** 10.3389/phrs.2026.1608732

**Published:** 2026-02-25

**Authors:** Maren Hintermeier, Kayvan Bozorgmehr, Nora Gottlieb, Amir Mohsenpour, Navina Sarma, Renke Biallas, Louise Biddle

**Affiliations:** 1 Section Health Equity Studies & Migration, University Hospital Heidelberg, Heidelberg, Germany; 2 Department of Population Medicine and Health Services Research, Bielefeld University, Bielefeld, Germany; 3 Robert Koch Institut, Berlin, Germany; 4 European Centre for Disease Prevention and Control (ECDC), Stockholm, Sweden; 5 Socio-Economic Panel (SOEP), DIW Berlin, Berlin, Germany

**Keywords:** COVID-19, health policy, interdisciplinary research, migrant populations, public health interventions

## Abstract

**Objectives:**

This study examines unintended consequences (UIC) of public health and social measures (PHSM) in camps and camp-like settings and assesses the pathways through which these UIC arise.

**Methods:**

We conducted a systematic review and conceptual analysis of UIC from PHSM aimed at preventing SARS-CoV-2 spread in these settings. PHSM were classified using the WHO taxonomy and the CONSEQUENT framework to analyse UIC pathways. The most frequent PHSM groups were: a) surveillance and response, b) social and physical distancing, and c) operational measures.

**Results:**

We identified 113 predominantly negative UIC impacting physical and mental health, healthcare access, economic stability, and social interactions. UIC occurred in both high- and low-income countries. Key mechanisms linking PHSM to UIC included mistrust, increased risk factors, lack of information, and uncertainty.

**Conclusion:**

This study reveals the complex interactions between PHSM and UIC and their broad mostly negative effects on marginalised populations. To reduce UIC in future health emergencies, they must be considered in pandemic planning with all stakeholders. Trust-building should be central in health interventions and PHSM design for more effective and equitable responses.

**Systematic Review Registration:**

https://www.crd.york.ac.uk/PROSPERO/view/CRD42022384673.

## Introduction

Research on COVID-19 has predominantly examined the effects of both pharmacological interventions (e.g., vaccines) and public health and social measures on epidemiological and clinical outcomes such as virus transmission and COVID-19-related illness and death [1, 2]. Public health and social measures designed to contain the COVID-19 pandemic always come with consequences, some intended, some unintended. There is no unanimously defined concept of unintended consequences, and notions and understandings vary depending on disciplines and fields of practice [3]. In this paper, we draw on the understanding of Jabeen (2018), who draws upon sociological literature to define intended outcomes as the product of explicit and formal formally organised action and procedures to achieve a certain result, purpose or desired change. Any other results of an intervention, understood as formally organized social action, which are not part of the initial intention are understood as the ‘unintended outcomes’ [4]. Jabeen (2018) further proposes a typology of unintended consequences organised by four dimensions, which we use to organize our analysis: Unintended consequences can be positive, negative or neutral and anticipated or unanticipated [4]. A further way to classify them is the “Consequences of Public Health Interventions” (CONSEQUENT) framework which also links unintended consequences to their underlying mechanisms [5]. With potential unintended consequences of public health and social measures becoming a matter of concern, some studies have focussed on consequences for mental health and economic productivity in the general population [6, 7]. Others have examined such consequences in specific contexts, e.g., related to travel restrictions [8] or in schools [9]. However, little attention has been paid to potential unintended consequences of public health and social measures in camps and camp-like settings for refugees, asylum seekers and internally displaced persons (IDPs). For the purposes of this study, ‘camps’ refer to longer-term formal settlements for displaced populations, while ‘camp-like settings’ denote temporary reception (e.g., transit centre) or collective accommodation centres [10]. Against the backdrop of already severe negative psychological effects of quarantine such as post-traumatic stress symptoms, anger and other stressors across all settings [6], this neglect is concerning given the additional unique circumstances of camps and camp-like settings, which include crowded living conditions, shared sanitary facilities, communal eating spaces, limited access to information and social support, and restrictions on freedom of movement [11, 12]. Additionally, the responsibility for residents lies with the authorities, who may sometimes have restricted capabilities to take action [12]. These contextual factors significantly increase the likelihood that public health and social measures result in unintended consequences. Moreover, refugees, asylum seekers and IDPs typically have a high burden of mental [13, 14] and physical illness [15, 16], resulting from hardships in their countries of origin, dangerous migration journeys and precarious living conditions in host countries, rendering them particularly vulnerable to the adverse effects of public health and social measures.

The aims of this study are to synthesise the existing evidence of the unintended consequences of public health and social measures implemented in camps and camp-like settings to prevent the spread of SARS-CoV-2, to analyse the pathways linking public health and social measures to their unintended consequences using the CONSEQUENT framework, and to reflect on the applicability of existing frameworks for understanding and mitigating unintended consequences.

## Methods

We performed a systematic review of the empirical literature on public health and social measures implemented in camps and camp-like settings for refugees, asylum seekers and IDPs in response to the COVID-19-pandemic. We used a conceptual analysis approach to explore and synthesise the unintended consequences of implemented public health and social measures in respective settings, guided by the “Consequences of Public Health Interventions” (CONSEQUENT) framework [5] to guide the classification of unintended consequences and the mechanisms through which they emerge. Methodologically, we built on a previous broad and comprehensive systematic review on COVID-19, displacement, and health [17] by developing a narrower set of inclusion and exclusion criteria (see below) to identify subsets of papers that report on public health and social measures and potential unintended consequences in camps and camp-like settings (first tier). A protocol containing details on how relevant literature was identified and extracted from the larger body of evidence of broader systematic reviews has been registered and published with PROSPERO: CRD42022384673 [18]. More recent literature was included using a search strategy specifically tailored to our research question, complementing the papers already identified within the previous systematic review (second tier).

### Search Strategy

A two-tiered search strategy was applied. First, all included studies (12/2019 to 11/2021) of a previous review [17] on COVID-19 health and health-related outcomes, including clinical outcomes such as infections, hospitalisation, mortality but also mental health outcomes or wellbeing and social determinants that affect health, among refugees and other migrant populations worldwide were screened with narrower inclusion criteria (i.e., population, setting, exposure). Second, an update search (12/2021 to 02/2023) in the WHO COVID-19 Research Database (representing 24 bibliographic databases [19]) and The Cochrane Library was conducted, using a search string created along the PECO scheme (see Supplementary Tables S1, S2a,b). Websites of the International Organization for Migration (IOM), the European Centre for Disease Prevention and Control (ECDC) and the European Public Health Association (EUPHA) were searched for further grey literature articles.

### Eligibility Criteria

We included empirical studies i) investigating refugees (including asylum seekers and IDPs) living in camps or camp-like settings such as reception and collective accommodation centres; and ii) reporting any type of public health and social measures based on the WHO taxonomy [20], such as individual measures, environmental, surveillance, response, or social and physical distancing measures; and iii) reporting any health-related outcomes (physical, mental, child, or maternal health, quality of life, social wellbeing) or social and economic outcomes. German, English and Spanish articles reporting empirical data using any research methodology were included from December 2019 until February 2023. Detailed inclusion and exclusion criteria are listed in the Supplementary Table S3.

### Screening Process

Titles, abstracts, and full-texts were screened by two reviewers independently. Conflicts were resolved by discussion or a third reviewer. Subsequently, we applied a third screening step to distinguish between studies reporting intended vs. unintended consequences of public health and social measures. Intended consequences were defined as any impact that reduced SARS-CoV-2 transmission or infection. Only the studies reporting unintended consequences were synthesised. We used the Covidence software for the screening process [21].

### Quality Appraisal

JBI checklists appropriate for the study design were used to assess the quality of the included studies [22]. For each study, the scores obtained from two independent ratings were averaged, and studies were grouped based on their scores and classified as high (100%–75% of full score), moderate (74%–50%), and low (<50%) quality studies. Rating discrepancies in case of considerably different scores (i.e., studies rated high by one reviewer and low by another) were resolved by discussion among the team. Questions marked “not applicable” were not included in the overall quality score to avoid artificially downgrading studies.

### Data Extraction and Management

We extracted bibliographic information (author, year, title), results of the quality assessment, study characteristics (country of study (according to the world bank country classifications by income level [23]), study period, migrant population, sample size and study design/methodology) as well as information on public health and social measures (i.e., description, period of implementation, target of measure (general vs. camp-specific), and context of implementation). To systematically describe unintended consequences, we used the framework of Jabeen [4] to assess their knowability (anticipated/unanticipated), value (positive/negative/neutral), distribution of effects (some/whole population), and temporality (simultaneously/after some time) as well as the relationship of unintended consequences with health, economic, and social outcomes (direct/indirect).

### Data Analysis and Presentation

After the extraction we classified the public health and social measures according to the WHO classification, and the unintended consequences according to the CONSEQUENT framework [5]. The framework categorises (unintended) consequences in multiple domains: health, health system, human and fundamental rights, acceptability and adherence, equity and equity-related, social and institutional, economic and resource-related, and ecological, whereas some consequences were not clearly assignable to single domains and therefore count into several domains, so that the percentages below exceed 100%. It also offers a set of potential mechanisms through which they emerge: bio-physiological mechanisms; (re-)action and behaviour change; perception, experience and assessment; available opportunities for (re-)action; environments and environmental exposure; social norms and practices; economic and market mechanisms; the functioning of systems and system components. Based on the frequency of measures identified, we split the public health and social measures into three main categories: a) surveillance and response measures in specific settings, including the protection of specific populations, b) social and physical distancing measures, and c) operational measures involving adaptations or closures of schools, health and social services, businesses, public spaces and administrative offices. We developed mosaic plots using the programming language R 4.3.2 to visualise the linkages between public health and social measures and unintended consequences (see Supplementary Chapter 4) and a diagram to visualise the cascading impacts of public health and social measures inspired by a framework of Carter et al. [24].

### Patient and Public Involvement

As this study is based on published literature, patient and public involvement was not applicable.

## Results

**FIGURE 1:**
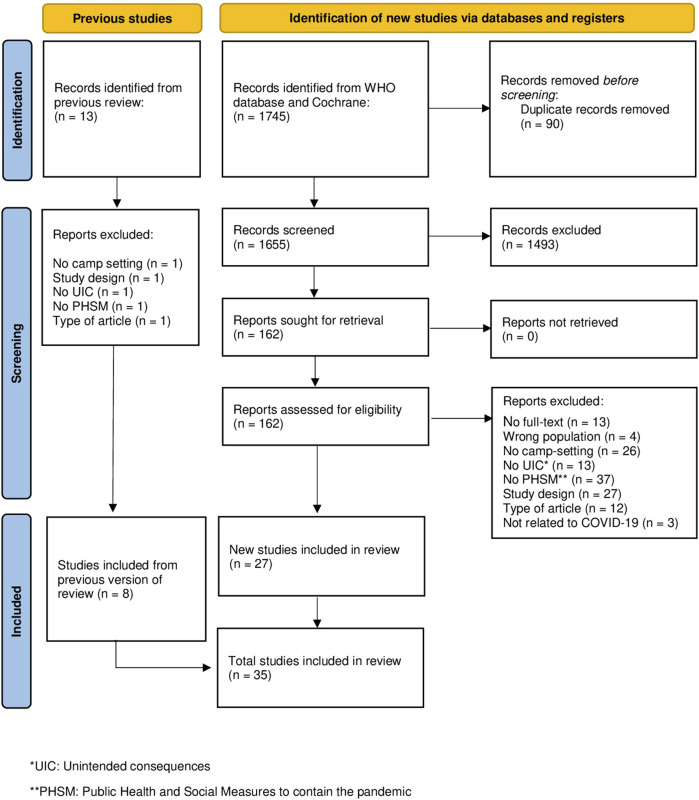
PRISMA Flow-Chart. UIC, Unintended consequences; PHSM, Public Health and Social Measures to contain the pandemic, studies published 12/2019-02/2023 (Germany).

Eight studies were included from the first-tier search. The second-tier search yielded 1,745 records, of which 1,655 titles and abstracts were screened after duplicate removal. Of these, 163 full-text articles were assessed for eligibility, and 27 studies were ultimately included, resulting in a total of 35 included studies (see Figure 1). Almost all studies were of high or medium quality, except for three studies, which were assessed as being of low quality due to insufficient contextual and methodological information [25], significant methodological limitations [26], and inconsistencies across several categories in the respective JBI checklist [27] (see Supplementary Tables S4a–f).

### Study Characteristics

The majority of the included studies were qualitative studies (n = 22; 62.9%), while ten (28.5%) were quantitative studies–comprising cross-sectional and cohort studies–and three (8.5%) were mixed-method studies (combining quantitative and qualitative research). In terms of migrant populations, 29 (82.9%) investigated refugees and asylum seekers, and three studies each (8.5%) examined IDPs and refugees in detention facilities. Geographically, the studies were conducted in 18 different countries, including five low-income countries (Burkina Faso, Mali, Rwanda, Somalia, Uganda), five low-middle-income countries (Bangladesh, Jordan, Kenya, Lebanon, Palestinian Territories (i.e., Westbank and Gaza)), and eight upper-middle and high-income countries (France, Germany, Greece, Iraq, Italy, Mexico, United Kingdom, and the United States) (see Table 1).

**TABLE 1:** Characteristics of the included studies, published 12/2019-02/2023 (Germany).

Author	Year	Country of study	Methodology	Migrant group	PSHM WHO classification of measure	Unintended consequences
Abu Hamad et al.	2022	Palestine (Gaza)	Mixed-methods	Refugees; IDPs	Social and physical distancing measures	Mental health impact, healthcare access
Ag Ahmed et al.	2021	Mali	Qualitative	IDPs	Social and physical distancing measures	Inability to look for work
Akhtar et al.	2021	Jordan	Quantitative	Refugees	Social and physical distancing measures	Mental health, limited access to resources
Apolot et al.	2023	Bangladesh	Mixed-methods	Refugees	Individual measures: using personal protective equipment; social and physical distancing measures	Environmental impact, PPE shortages, PPE is not adapted to gender and culture; improved implementation of measure; increased trust in health staff
Asoni et al.	2023	USA	Qualitative	ICE-detainees	Social and physical distancing measures	Hunger strike
Berg	2022	Germany	Qualitative	Refugees; asylum seekers	Social and physical distancing measures	Social and healthcare access impaired; information precarity; impaired access to online education
Berg et al.	2022	NJ/USA	Qualitative	ICE-detainees	Social and physical distancing measures	No access to due process
Biddle et al.	2021	Germany	Qualitative	Asylum seekers and refugees	Social and physical distancing measures	Mental health; delayed notification of positive tests
Boehme et al.	2022	Germany; Greek islands; Kenya	Qualitative	Refugees	Social and physical distancing measures	Mistrust; mental health; conflicts
Can Collado et al.	2021	Mexico	Qualitative	Other: Mixed migrant population	Social and physical distancing measures	Delays in immigration permit procedures; mental health
Chowdury et al.	2022	Bangladesh	Qualitative	Other: Healthcare workers	Individual measures; social and physical distancing measures	Financial opportunities; unemployment; physical/mental health; healthcare/services access; misinformation
Cortez et al.	2021	Uganda	Qualitative	Refugees	Individual measures	Nutrition, health and hygiene
Crouzet et al.	2022	France	Qualitative	Other: Homeless people including foreign-born	Social and physical distancing measures	Uncertainty about asylum procedure
Cruz Pineiro & Ibarra	2022	Mexico	Qualitative	Asylum seekers	Biological measures; social and physical distancing measures	Vaccination without clear informed consent; failure of migration project; individuals leaving shelter
DaMosto et al.	2021	Italy	Qualitative	People living in reception centres	Individual measures; social and physical distancing measures; surveillance and response measures	Healthcare access; mental health; increased infection risk; information flow; reduced social capital; impact on migration project; job loss; no integration activities; lack of trust
Filippi et al.	2021	Italy	Qualitative	Asylum seekers and refugees in reception centres	Social and physical distancing measures	Higher infection risk; impact of migration project; non-compliance
Filosi et al.	2022	Italy	Qualitative	Asylum seekers	Social and physical distancing measures; other	Distrust; mental health; loss of economic opportunities; non-affordability of food; education; lack of social interaction
Ghaddar et al.	2023	Lebanon	Qualitative	Refugees; other: Healthcare workers	Surveillance and response measures	Refugees refused to test for COVID-19 for fear of deportation
Hamdan et al.	2021	Palestine Territories	Quantitative	Refugees	Social and physical distancing measures	Effects on mobility; mental health
Infante et al.	2022	Mexico	Qualitative	Other: Staff members and volunteers, health service providers, authorities of local health services, and others	Individual measures	Healthcare access; complete closure of one migrant shelter
Jahn et al.	2022	Germany	Quantitative	Asylum seekers	Social and physical distancing measures; surveillance and response measures	Increased infection risk; conflicts
Kizilhan et al.	2020	Iraq	Quantitative	Refugees	Social and physical distancing measures	Mental health decline
Kondilis et al.	2021	Greece	Quantitative	Refugees and asylum seekers	Social and physical distancing measures	Increased infection risk
Korobkova et al.	2022	Multiple countries	Quantitative	Refugees; asylum seekers; IDPs	Social and physical distancing measures	Heightened exposure to violence, neglect, abuse; healthcare access in different countries; delay in asylum claim processing; impact on livelihoods and income; impacts on psychological wellbeing; food shortages for children
Meyer et al.	2022	Rwanda	Qualitative	Refugees; focus groups also included health providers, community members and parents	Social and physical distancing measures	Increased risky behaviours; healthcare access & treatment delay/omission; mental health decline; lack of employment and livelihood opportunities, transactional sex
Mistry et al.	2021	Bangladesh	Quantitative	Refugees	Social and physical distancing measures	Loneliness; healthcare access
Mumin et al.	2022	Somalia	Quantitative	IDPs	Social and physical distancing measures	Decreased income; mental health; healthcare access; reductions in food distribution
Nwadiuko et al.	2023	USA	Quantitative	ICE-detainees	Surveillance and response measures	Solitary confinement is assumed to lead to well-known negative consequences
Ozer et al.	2022	Burkina Faso	Qualitative	IDPs	Social and physical distancing measures	Decrease in amount, quality and frequency of assistance, incl. Food supplies; no/lower income
Rangel Gomez et al.	2023	Mexico	Qualitative	Other: People in charge, managers, coordinators, shelter directors from 22 migrant shelters	Social and physical distancing measures; surveillance and response measures	Mass quarantine; discouragement in reporting symptoms; protests; isolation from family; several shelters forced to close or operate behind closed doors
Reynolds et al.	2022	Mexico	Qualitative	Asylum seekers; other: Healthcare professionals	Social and physical distancing measures; surveillance and response measures	Negative effects on mental health; mistrust; quarantine for non-infectious individuals
Stein et al.	2022	Uganda	Mixed-methods	Refugees	Social and physical distancing measures	Lack of money; food insecurity
Stillman et al.	2022	Jordan	Quantitative	Refugees	Social and physical distancing measures	Reduction of income (also post lockdown); unemployment
Tschalaer	2022	United Kingdom	Qualitative	Refugees; asylum seekers; other: Social/charity workers	Social and physical distancing measures	Increased risk of sexual violence; loss of community/family; mental health impact

PHSM, Public Health and Social Measures to contain the pandemic; IDPs, Internally displaced persons.

### Overview of Public Health and Social Measures and Associated Unintended Consequences

We found public health and social measures related to different categories of the WHO taxonomy, namely, individual measures (such as using personal protective equipment (PPE) or mask wearing) [27–31], surveillance and response measures (i.e., detecting and isolating cases) [12, 28, 30, 32–38], social and physical distancing measures [12, 25, 26, 28–32, 34, 35, 37–56], and less frequently environmental and biological measures [46] (which are not part of this analysis). Social and physical distancing measures were the biggest group and the main focus of our analysis. This category contains various measures including domestic travel restrictions including stay-at-home orders and mobility restrictions, measures for special populations, measures for offices, businesses, institutions and operations, school closures, and the like.

Across all these public health and social measures we identified 113 unintended consequences. 33.6% (n = 38) of unintended consequences related to health consequences including psychosocial health and wellbeing, and physical health and health behaviours. 23.0% (n = 26) referred to the health system, namely, to access to, utilisation of, and quality of health services, and acceptability and adherence to health services. Economic and resource-related unintended consequences accounted for 15.0% (n = 17), while 8.0% (n = 9) were related to human and fundamental rights, specifically autonomy, self-determination, and privacy. Finally, 28.3% (n = 32) of the unintended consequences were related to social and institutional factors, including the legal and political system, social cohesion and wellbeing, safety, security, and crime, conditions of daily living, and education and development. One unintended consequence referred to an ecological consequence in a low-middle income country due to the use of PPE [28]. Table 2 provides a detailed description of each unintended consequence, as noted in the primary studies.

**TABLE 2:** Characteristics of the included studies by unintended consequences, studies published 12/2019-02/2023 (Germany).

Author	Year	Country of study	Methodology	Migrant group	PSHM WHO classification of measure	Description of unintended consequences (UIC)	Value of the UIC	Analysis of UIC as a goal of the study	Study quality^a^
Abu Hamad et al.	2022	Palestine (Gaza)	Mixed-methods	Refugees; IDPs	Social and physical distancing measures: Domestic travel (incl. Stay-at-home orders and movement restrictions)	Mental health impact	Negative	Yes	Moderate
Abu Hamad et al.	2022	Palestine (Gaza)	Mixed-methods	Refugees; IDPs	Social and physical distancing measures: offices, businesses, institutions and operations	Access to care further compromised	Negative	No	Moderate
Abu Hamad et al.	2022	Palestine (Gaza)	Mixed-methods	Refugees; IDPs	Social and physical distancing measures: School measures	Mental health impact	Negative	Yes	Moderate
Ag Ahmed et al.	2021	Mali	Qualitative	IDPs	Social and physical distancing measures: Domestic travel (incl. Stay-at-home orders and movement restrictions)	Inability to look for work	Negative	No	Moderate
Akhtar et al.	2021	Jordan	Quantitative	Refugees	Social and physical distancing measures	Greater decrease in PTSD symptom severity in participants with PTSD symptoms prior to the pandemic	Positive	No	Moderate
Akhtar et al.	2021	Jordan	Quantitative	Refugees	See above	Limited access to resources (refugees confined to the camp and reliant on the limited supplies made available to them by camp authorities and NGOs operating within the camp)	Negative	No	Moderate
Apolot et al.	2023	Bangladesh	Mixed-methods	Refugees	Individual measures: using personal protective equipment	Frequent breakdown of incinerators due to high quantities of waste	Negative	No	High
Apolot et al.	2023	Bangladesh	Mixed-methods	Refugees	See above	Excessive use in the early phase of the pandemic exacerbated PPE shortages and waste disposal challenges	Negative	No	High
Apolot et al.	2023	Bangladesh	Mixed-methods	Refugees	See above	PPE is not adapted to gender and culture; e.g., scrubs have no head coverings for women; masks have only ear bands, which cannot be used by women wearing hijab	Negative	No	High
Apolot et al.	2023	Bangladesh	Mixed-methods	Refugees	Social and physical distancing measures: supervision visits to control measures in place at refugee camp	Supposedly, the aim of the measure was just quality control; but it was said to also improve implementation of measures	Positive	No	High
Apolot et al.	2023	Bangladesh	Mixed-methods	Refugees	Surveillance and response measures: Detecting and isolating cases	The measure is aimed at protecting patients/visitors from infection from positive staff, but also generates trust in the health staff and health facilities as a “safe place”	Positive	No	High
Asoni et al.	2023	USA	Qualitative	ICE-detainees	Social and physical distancing measures: Collective quarantine and suspension of visitors in detention facility	Hunger strike	Unclear	Yes	High
Berg	2022	Germany	Qualitative	Refugees; asylum seekers	Social and physical distancing measures: offices, businesses, institutions and operations	social workers no longer able to access facilities; impaired access to German social infrastructure	Negative	Yes	Moderate
Berg	2022	Germany	Qualitative	Refugees; asylum seekers	See above	Information precarity due to inaccessibility of internet: No social contact, no access to information	Negative	Yes	Moderate
Berg	2022	Germany	Qualitative	Refugees; asylum seekers	Social and physical distancing measures: School measures	Impaired access to online education (both schooling and language courses) due to inaccessibility of internet	Negative	Yes	Moderate
Berg	2022	Germany	Qualitative	Refugees; asylum seekers	See above	Lack of access to information facilitates spread of misinformation, in turn leading to low trust in institutions and officials	Negative	Yes	Moderate
Berg	2022	Germany	Qualitative	Refugees; asylum seekers	Social and physical distancing measures: Multiple collective quarantines	Information precarity due to inaccessibility of internet: No social contact, no access to information	Negative	Yes	Moderate
Berg et al.	2022	NJ/USA	Qualitative	ICE-detainees	Social and physical distancing measures: Suspension of visitors in detention facility	No access to due process (see details below)	Negative	Yes	High
Biddle et al.	2021	Germany	Qualitative	Asylum seekers and refugees	Social and physical distancing measures: offices, businesses, institutions and operations	Loss of daily structure leading to heavier psychosocial stress	Negative	No	Moderate
Biddle et al.	2021	Germany	Qualitative	Asylum seekers and refugees	Surveillance and response measures: Detecting and isolating cases	Delayed notification of camp manager and infected person in case of positive test	Negative	No	Moderate
Boehme et al.	2022	Germany; Greek islands; Kenya	Qualitative	Refugees	Social and physical distancing measures: Facility-wide quarantine and testing	Mistrust in camp administration (inconsistencies, e.g., testing results quicker than expected, one person received positive test without being tested, etc.)	Negative	No	Moderate
Boehme et al.	2022	see above	Qualitative	Refugees	See above	Isolation and unrest, boredom	Negative	No	Moderate
Boehme et al.	2022	see above	Qualitative	Refugees	See above	Conflicts among inhabitants and between inhabitants and staff increased (over pandemic rules)	Negative	No	Moderate
Boehme et al.	2022	see above	Qualitative	Refugees	See above	Fear of infection (within the camp i.e., camp perceived as unsafe)	Negative	No	Moderate
Boehme et al.	2022	see above	Qualitative	Refugees	See above	Growing mistrust among inhabitants	Negative	No	Moderate
Boehme et al.	2022	see above	Qualitative	Refugees	See above	Conflict with local population	Negative	No	Moderate
Boehme et al.	2022	see above	Qualitative	Refugees	See above	Insecurity about returning to facility	Negative	No	Moderate
Can Collado et al.	2021	Mexico	Qualitative	Other: Mixed migrant population	Social and physical distancing measures: offices, businesses, institutions and operations	Delays in immigration permit procedures	Negative	No	Moderate
Can Collado et al.	2021	Mexico	Qualitative	Other: Mixed migrant population	Surveillance and response measures: Detecting and isolating cases	Mental health consequences	Negative	No	Moderate
Chowdury et al.	2022	Bangladesh	Qualitative	Other: Healthcare workers	Individual measures: wearing a mask	Financial opportunities for women in mask making	Positive	No	High
Chowdury et al.	2022	Bangladesh	Qualitative	Other: Healthcare workers	Social and physical distancing measures: Domestic travel (incl. Stay-at-home orders and movement restrictions)	Male unemployment	Negative	Yes	High
Chowdury et al.	2022	Bangladesh	Qualitative	Other: Healthcare workers	See above	Increase in the incidence of sexual and gender-based violence	Negative	Yes	High
Chowdury et al.	2022	Bangladesh	Qualitative	Other: Healthcare workers	Social and physical distancing measures: Offices, businesses, institutions and operations	Decreased service availability	Negative	Yes	High
Chowdury et al.	2022	Bangladesh	Qualitative	Other: Healthcare workers	See above	Decreased utilisation of services, inability for outreach and communication in public meetings, inability to make referrals	Negative	Yes	High
Chowdury et al.	2022	Bangladesh	Qualitative	Other: Healthcare workers	See above	Confusion and misinformation about the availability of services during the lockdown period, resulting in decreased utilisation	Negative	Yes	High
Chowdury et al.	2022	Bangladesh	Qualitative	Other: Healthcare workers	See above	Issues around confidentiality as women had to use partners’ phones for counselling and home visits were no longer possible	Negative	Yes	High
Cortez et al.	2021	Uganda	Qualitative	Refugees	Individual measures: Performing hand hygiene	Queuing for cash assistance and soap meant physical fatigue and exposure to heat	Negative	No	Low
Cortez et al.	2021	Uganda	Qualitative	Refugees	See above	Rationing water for handwashing reduced water amounts for drinking and bathing, with impacts on nutrition, health and hygiene	Negative	No	Low
Crouzet et al.	2022	France	Qualitative	Other: Homeless people including foreign-born	Social and physical distancing measures	Uncertainty about asylum procedure	Negative	No	Moderate
Cruz Pineiro & Ibarra	2022	Mexico	Qualitative	Asylum seekers	Biological measures	Vaccination without clear informed consent	Negative	No	High
Cruz Pineiro & Ibarra	2022	Mexico	Qualitative	Asylum seekers	Social and physical distancing measures: International travel measures	Failure of migration project	Negative	No	High
Cruz Pineiro & Ibarra	2022	Mexico	Qualitative	Asylum seekers	Social and physical distancing measures: strict schedules (e.g., for meals) and rules	Individuals leaving shelter because of highly restrictive policies	Negative	No	High
DaMosto et al.	2021	Italy	Qualitative	People living in reception centres	Individual measures: using personal protective equipment (PPE)	Lack of PPE and overcrowding led to 1) longer and more severe curfew; 2) interruption of HC provision via volunteer clinics; 3) higher exposure and stress for residents and staff; 4) higher workload for staff, who try to organize PPE individually	Negative	Yes	Moderate
DaMosto et al.	2021	Italy	Qualitative	See above	Individual measures: wearing a mask	Impact on the transmission of verbal and non-verbal messages, difficulties in engaging with camp residents	Negative	Yes	Moderate
DaMosto et al.	2021	Italy	Qualitative	See above	Social and physical distancing measures	High infection rates (i.e., increased infection risk)	Negative	Yes	Moderate
DaMosto et al.	2021	Italy	Qualitative	See above	Social and physical distancing measures: Domestic travel (incl. Stay-at-home orders and movement restrictions)	The strict lockdown led to reduced social capital and community networks, mental health impact, re-traumatization, and facilitated “intensified… level(s) of (interpersonal and institutional) discrimination”	Negative	Yes	Moderate
DaMosto et al.	2021	Italy	Qualitative	See above	Social and physical distancing measures: offices, businesses, institutions and operations	Impact on migration project: Suspension of asylum procedures, impossibility to renew residence permit, loss of job and income, inability to send money home, uncertainty about future. Also, more hazardous employment, incl. Survival prostitution	Negative	Yes	Moderate
DaMosto et al.	2021	Italy	Qualitative	See above	See above	Low/no access to healthcare (also suspension of services), including screening and testing	Negative	Yes	Moderate
DaMosto et al.	2021	Italy	Qualitative	See above	See above	Unavailability of healthcare increases workload and responsibilities of staff, especially social workers who have to provide counselling	Negative	Yes	Moderate
DaMosto et al.	2021	Italy	Qualitative	See above	See above	The lockdown made integration activities, such as language classes, internships, etc. impossible for refugees and asylum seekers	Negative	Yes	Moderate
DaMosto et al.	2021	Italy	Qualitative	See above	Social and physical distancing measures: Transfer of individuals with special protection needs	Higher COVID-19 risk, lack of credibility of public health messaging due to overcrowding, lack of possibilities for social distancing even for vulnerable people	Negative	Yes	Moderate
DaMosto et al.	2021	Italy	Qualitative	See above	Surveillance and response measures: Detecting and isolating cases	Conflicting public health messaging and response led to conspiracy theories, lack of trust, non-compliance	Negative	Yes	Moderate
Filippi et al.	2021	Italy	Qualitative	Asylum seekers and refugees in reception centres	Social and physical distancing measures: Domestic travel (incl. Stay-at-home orders and movement restrictions)	Lockdown is first interpreted as a direct, racist attack on asylum seekers and refugees; Then, due to the camp conditions making protective measures impossible, authors describe “lack of interest” among staff, non-compliance, or “abandonment”, which eventually leads to higher risk of infection, failure to achieve intended effect	Negative	Yes	Moderate
Filippi et al.	2021	Italy	Qualitative	See above	Social and physical distancing measures: offices, businesses, institutions and operations	Impact on migration project: Suspension of asylum procedures, impossibility to renew residence permit, loss of job and income, inability to send money home, uncertainty about future	Negative	Yes	Moderate
Filippi et al.	2021	Italy	Qualitative	See above	Social and physical distancing measures: Curfew	Higher infection risk	Negative	Yes	Moderate
Filosi et al.	2022	Italy	Qualitative	Asylum seekers	Other	Distrust (of other residents) and anxiety, as not everyone followed the rules	Negative	No	Moderate
Filosi et al.	2022	Italy	Qualitative	Asylum seekers	Social and physical distancing measures: offices, businesses, institutions and operations	Loss of economic opportunities as job interviews were cancelled and contracts not renewed	Negative	No	Moderate
Filosi et al.	2022	Italy	Qualitative	Asylum seekers	Social and physical distancing measures: Collective quarantine (of facility)	Non-affordability of food, higher prices in the closer supermarkets	Negative	No	Moderate
Filosi et al.	2022	Italy	Qualitative	Asylum seekers	See above	Constant presence of other people in common rooms, inability to find a place to study	Negative	No	Moderate
Filosi et al.	2022	Italy	Qualitative	Asylum seekers	See above	Inability to purchase one’s own food, have autonomy over nourishment	Negative	No	Moderate
Filosi et al.	2022	Italy	Qualitative	Asylum seekers	See above	Delayed emancipation from facility due to the lack of social relations and economic opportunities	Negative	No	Moderate
Filosi et al.	2022	Italy	Qualitative	Asylum seekers	See above	Lack of social interaction & contact inside and outside the facility, negative impacts on mental health (boredom & loneliness)	Negative	No	Moderate
Ghaddar et al.	2023	Lebanon	Qualitative	Refugees; other: Healthcare workers	Surveillance and response measures: Detecting and isolating cases	Refugees refused to test for COVID-19 for fear of deportation if they tested positive	Negative	No	High
Hamdan et al.	2021	Palestine Territories	Quantitative	Refugees	Social and physical distancing measures: Domestic travel (incl. Stay-at-home orders and movement restrictions)	Effects on mobility and self-care dimensions of health-related quality of life (HRQoL)	Negative	Yes	High
Infante et al.	2022	Mexico	Qualitative	Other: Staff members and volunteers, health service providers, authorities of local health services, and others	Individual measures	Closure of new admissions (decision of camp staff) and complete closure of one migrant shelter (mandated by government) due to impossibility of adhering to guidelines	Negative	No	Moderate
Infante et al.	2022	Mexico	Qualitative	See above	Social and physical distancing measures: offices, businesses, institutions and operations	Decreased ability to respond to other health needs	Negative	No	Moderate
Infante et al.	2022	Mexico	Qualitative	See above	See above	Lower availability of medical services	Negative	No	Moderate
Jahn et al.	2022	Germany	Quantitative	Asylum seekers	Social and physical distancing measures: Collective quarantine (of facility)	Higher SARS-CoV-2 attack rates compared to sites applying conventional management strategies; conflicts within the camps are reported in connection with mass quarantine	Negative	Yes	High
Jahn et al.	2022	Germany	Quantitative	Asylum seekers	Surveillance and response measures: Detecting and isolating cases	Higher SARS-CoV-2 attack rates in outbreaks implementing mass testing compared to sites implementing targeted testing of close contacts or only symptomatic inhabitants	Negative	Yes	High
Kizilhan et al.	2020	Iraq	Quantitative	Refugees	Social and physical distancing measures: Collective quarantine (of facility)	Mental health decline	Negative	No	Low
Kondilis et al.	2021	Greece	Quantitative	Refugees and asylum seekers	Social and physical distancing measures: Collective quarantine (of facility)	Increased infection risk	Negative	No	High
Korobkova et al.	2022	Multiple countries	Quantitative	Refugees; asylum seekers; IDPs	Social and physical distancing measures	Heightened exposure to violence, neglect, abuse, and exploitation for children	Negative	No	Low
Korobkova et al.	2022	Multiple countries	Quantitative	Refugees; asylum seekers; IDPs	Social and physical distancing measures: Domestic travel (incl. Stay-at-home orders and movement restrictions)	worse access to healthcare for refugees in Turkey and DRC	Negative	No	Low
Korobkova et al.	2022	Multiple countries	Quantitative	Refugees; asylum seekers; IDPs	See above	worse access to healthcare in Uganda	Negative	No	Low
Korobkova et al.	2022	Multiple countries	Quantitative	Refugees; asylum seekers; IDPs	Social and physical distancing measures: offices, businesses, institutions and operations	Delay in asylum claim processing	Negative	No	Low
Korobkova et al.	2022	Multiple countries	Quantitative	Refugees; asylum seekers; IDPs	See above	Inability to access vaccinations	Negative	No	Low
Korobkova et al.	2022	Multiple countries	Quantitative	Refugees; asylum seekers; IDPs	See above	Impact on livelihoods and income	Negative	No	Low
Korobkova et al.	2022	Multiple countries	Quantitative	Refugees; asylum seekers; IDPs	See above	Impacts on psychological wellbeing	Negative	No	Low
Korobkova et al.	2022	Multiple countries	Quantitative	Refugees; asylum seekers; IDPs	See above	Food shortages for children	Negative	No	Low
Meyer et al.	2022	Rwanda	Qualitative	Refugees; focus groups also included health providers, community members and parents	Social and physical distancing measures: offices, businesses, institutions and operations	Increased risk of unprotected sex, unplanned pregnancies, substance consumption, and STIs	Negative	Yes	Moderate
Meyer et al.	2022	Rwanda	Qualitative	See above	See above	Delays in/omission of medical treatment for general sexual related health questions, pregnancy and STI-screening, contraceptive counselling and the like (access to healthcare)	Negative	Yes	Moderate
Meyer et al.	2022	Rwanda	Qualitative	See above	See above	Increased anxiety and risky behaviours	Negative	Yes	Moderate
Meyer et al.	2022	Rwanda	Qualitative	See above	See above	Different treatment plans during COVID-19 for the same illness someone suffered and was treated before the pandemic; some services were no longer offered	Negative	Yes	Moderate
Meyer et al.	2022	Rwanda	Qualitative	See above	Social and physical distancing measures: School measures	Increase in risky sexual behaviour, sexual violence, and unwanted pregnancies	Negative	Yes	Moderate
Meyer et al.	2022	Rwanda	Qualitative	See above	Social and physical distancing measures: Inability to leave the camp & school closure	Lack of employment and livelihood opportunities, transactional sex	Negative	Yes	Moderate
Meyer et al.	2022	Rwanda	Qualitative	See above	Surveillance and response measures: Detecting and isolating cases	Delays in/omission of treatment of COVID-19 disease (access to healthcare)	Negative	Yes	Moderate
Mistry et al.	2021	Bangladesh	Quantitative	Refugees	Social and physical distancing measures	Loneliness	Negative	No	High
Mistry et al.	2021	Bangladesh	Quantitative	Refugees	Social and physical distancing measures: Domestic travel (incl. Stay-at-home orders and movement restrictions)	Difficulties in accessing medicine and routine medical care	Negative	No	High
Mumin et al.	2022	Somalia	Quantitative	IDPs	Social and physical distancing measures: Gatherings, businesses and services	Decreased income due to closure of markets	Negative	Yes	High
Mumin et al.	2022	Somalia	Quantitative	IDPs	See above	Negative mental health implications	Negative	Yes	High
Mumin et al.	2022	Somalia	Quantitative	IDPs	See above	Reductions in food distribution	Negative	Yes	High
Mumin et al.	2022	Somalia	Quantitative	IDPs	See above	Reduction of health service provision	Negative	Yes	High
Nwadiuko et al.	2023	USA	Quantitative	ICE-detainees	Surveillance and response measures: Detecting and isolating cases	Solitary confinement is assumed to lead to well-known negative consequences	Negative	No	Moderate
Ozer et al.	2022	Burkina Faso	Qualitative	IDPs	Social and physical distancing measures	Decrease in amount, quality and frequency of assistance, incl. Food supplies	Negative	Yes	Moderate
Ozer et al.	2022	Burkina Faso	Qualitative	IDPs	See above	Relocation process of the camp was slowed down	Negative	Yes	Moderate
Ozer et al.	2022	Burkina Faso	Qualitative	IDPs	See above	No/lower income	Negative	Yes	Moderate
Rangel Gomez et al.	2023	Mexico	Qualitative	Other: People in charge, managers, coordinators, shelter directors from 22 migrant shelters	Social and physical distancing measures: Collective quarantine (of facility)	Discouraging institutions from notifying suspected cases of the health Jurisdiction as it may lead to quarantine for entire institution	Negative	No	Moderate
Rangel Gomez et al.	2023	Mexico	Qualitative	see above	Surveillance and response measures: Detecting and isolating cases	Migrants were discouraged from reporting symptoms due to a lack of clarity and transparency in protocol compliance	Negative	No	Moderate
Rangel Gomez et al.	2023	Mexico	Qualitative	see above	see above	Isolation from friends and family	Negative	No	Moderate
Rangel Gomez et al.	2023	Mexico	Qualitative	see above	see above	Protests, sit-ins, riots, plots, and uprisings	Negative	No	Moderate
Rangel Gomez et al.	2023	Mexico	Qualitative	see above	see above	several shelters forced to close or operate behind closed doors	Negative	No	Moderate
Reynolds et al.	2022	Mexico	Qualitative	Asylum seekers; Other: Healthcare professionals	Social and physical distancing measures: offices, businesses, institutions and operations	Negative effects on mental health	Negative	Yes	Moderate
Reynolds et al.	2022	Mexico	Qualitative	See above	See above	Increased uncertainty exacerbated mental health conditions	Negative	No	Moderate
Reynolds et al.	2022	Mexico	Qualitative	See above	Surveillance and response measures: Detecting and isolating cases	Distrust towards healthcare providers	Negative	No	Moderate
Reynolds et al.	2022	Mexico	Qualitative	See above	See above	Quarantining of non-infectious individuals	Negative	No	Moderate
Stein et al.	2022	Uganda	Mixed-methods	Refugees	Social and physical distancing measures: Offices, businesses, institutions and operations	Lack of money, food insecurity	Negative	No	High
Stein et al.	2022	Uganda	Mixed-methods	Refugees	Social and physical distancing measures: School measures	Food shortages due to children being at home and not at school	Negative	No	High
Stillman et al.	2022	Jordan	Quantitative	Refugees	Social and physical distancing measures: Domestic travel (incl. Stay-at-home orders and movement restrictions)	80% reduction in per-adult income during lockdown, only ca. 20% of households had someone in employment	Negative	No	High
Stillman et al.	2022	Jordan	Quantitative	Refugees	See above	77% reduction in per-adult income post lockdown	Negative	No	High
Tschalaer	2022	United Kingdom	Qualitative	Refugees; asylum seekers; other: Social/charity workers	Social and physical distancing measures: Domestic travel (incl. Stay-at-home orders and movement restrictions)	Increased risk of sexual violence	Negative	No	High
Tschalaer	2022	United Kingdom	Qualitative	See above	Social and physical distancing measures: offices, businesses, institutions and operations	Loss of community/family, mental health impact	Negative	No	High
Tschalaer	2022	United Kingdom	Qualitative	See above	See above	Loss of spaces of “belonging”, feelings of loneliness and isolation	Negative	No	High

^a^ Quality assessed based on JBI checklists.

### Conceptual Linkage Between Public Health and Social Measures and Unintended Consequences

**FIGURE 2:**
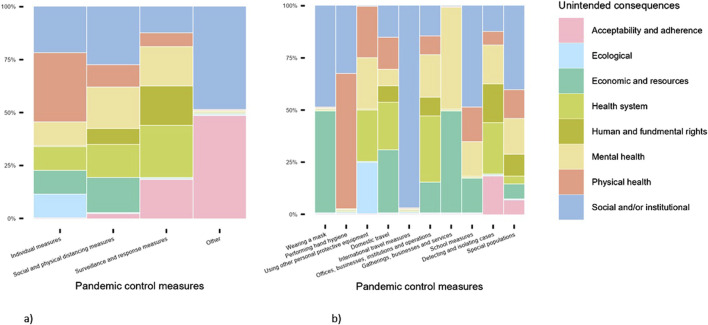
Proportion of unintended consequences by pandemic control measures with broad **(a)** and granular aggregation **(b)** (unweighted), studies published 12/2019-02/2023 (Germany).

Figure 2 shows the distribution of unintended consequences by public health and social measures. The graph highlights that unintended physical health consequences were primarily reported in the context of individual measures (Figure 2a), particularly hand hygiene and the use of PPE (Figure 2b). Measures related to gatherings, businesses, and services were reported as having a significant impact on mental health, while social and physical distancing measures were primarily reported with respect to health system-related unintended consequences (Figure 2a). Two additional key findings become evident from the illustration. First, international travel restrictions are reported to have a significant impact on social and institutional outcomes, such as increased exposure to crime and delays in or failures of migration projects (Figure 2b). Second, detecting and isolation measures are the primary reported drivers of unintended consequences, particularly regarding human and fundamental rights as well as the acceptability and adherence to health services (Figure 2b).

### Pathways Between Public Health and Social Measures and Unintended Consequences

**FIGURE 3:**
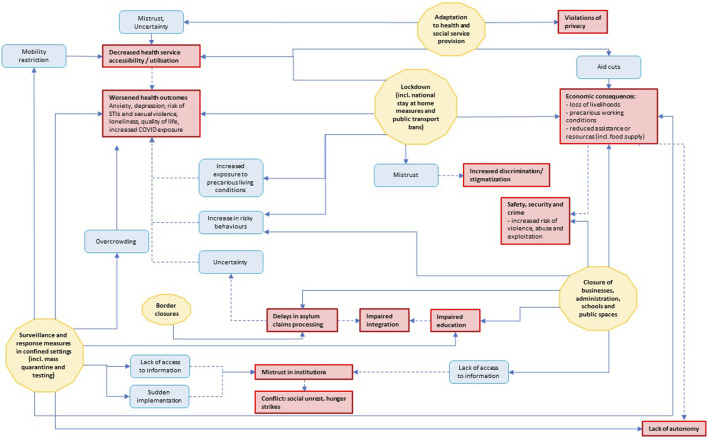
Pathways and mechanisms between public health and social measures and unintended consequences. Yellow polygon: public health and social measures; Blue squares: mechanisms; Red shapes: unintended consequences across all three groups. Solid lines: directs effects; dashed lines: indirect effects. Studies published 12/2019-02/2023 (Germany).

This section integrates the three main public health and social measure categories (subsections *Surveillance and Response Measures in Specific Settings, Social and Physical Distancing Measures, Operational Measures*) with their associated unintended consequences. Figure 3 provides an overarching synthesis of the pathways through which public health and social measures give rise to unintended consequences across all three categories, distinguishing direct and indirect pathways (solid and dashed arrows) and highlights underlying mechanisms (blue squares) linking public health and social measures (yellow polygon) to unintended consequences (red shapes). Guided by this integrative framework, the following subsections examine each public health and social measure in detail. More detailed pathway diagrams for each public health and social measure group are provided in the Supplementary Figures S2a–c.

#### Surveillance and Response Measures in Specific Settings

Within the category of surveillance and response measures, the most frequently described public health and social measures were mass quarantine and isolation, as well as mandatory testing in closed settings such as refugee camps, reception centres or detention centres [12, 26, 28, 30, 32, 34, 35, 37, 38, 41–44, 46–48, 50]. We identified 38 unintended consequences attributable to this group of measures.

Direct unintended consequences resulting from this public health and social measure group included a deterioration in health outcomes–particularly mental health [32, 33, 36, 44, 48] – and an increased risk of SARS-CoV-2 infection [12, 26, 34, 44, 47]. Other immediate effects involved a reduction or even lack of autonomy [37, 42, 44, 48], as perceived by the asylum seekers and refugees residing in these facilities, as well as economic consequences and hardships [35, 37, 44, 48]. Health outcomes were further worsened by crowded living conditions which was triggered or amplified by mass quarantine. The sudden implementation of measures, along inadequate preparation and communication with the affected population, led to mistrust of institutions and authorities, which ultimately gave rise to conflicts and hunger strikes in some facilities [30, 37, 38, 41, 44]. The mobility restrictions imposed as part of the mass quarantine affected access to health services and prevented residents from seeking medical care outside the refugee camps [28, 30, 35]. In some cases, no health services were provided in camps and camp-like settings and aid organisations were denied access due to quarantine measures, leaving refugees without access to healthcare [28, 35].

Even when mechanisms were not explicitly analysed, many papers emphasised the contextual conditions under which public health and social measures were implemented and in which unintended consequences emerged. This underscores the importance of considering pre-existing restrictions in camp and camp-like settings–such as limited access to services, lack of internet access, insufficient communication and education, and geographically deprived locations–when assessing mechanisms and their effects.

#### Social and Physical Distancing Measures

The main measures reported in the category of social and physical distancing measures included closure or ban of public transport [25, 51], stay-at-home orders [25, 39, 47, 56, 57], general lockdown measures (often not further specified) [25, 29, 30, 40, 45, 49, 51, 53, 55], and border closures [46]. In Figure 3, we divided these measures into two groups: national lockdown measures (including national stay-at-home orders and public transport bans) and international border closures. The latter, which was reported only once, directly impacted and delayed asylum applications. This, in turn, generated uncertainty as a mechanism indirectly contributing to a deterioration in health [46].

In camps and camp-like settings, lockdown measures contributed to negative health outcomes, such as food insecurity [53], deteriorating mental health [45, 49, 51], and an increased risk of infection [30]. They also reduced access to healthcare [25, 29, 51] and caused significant economic impacts, including job and income loss [29, 35, 55], and decreased resources and support [30, 53], including food supply [46]. In such settings, stay-at-home orders had additional social and institutional consequences, such as a heightened risk of gender-based and domestic violence, neglect, abuse, and exploitation of children [25, 56]. These lockdown-induced effects were driven by increased exposure to unstable living conditions and the emergence in risky behaviours, both of which exacerbated health problems. In addition, the measures contributed to heightened mistrust, which in turn led to greater perceived discrimination and stigmatisation at both institutional and interpersonal levels [30, 47].

#### Operational Measures

Unintended consequences resulting from public health and social measures classified as operational measures encompassed adaptations and closures of five key sectors: schools and education, health and social services, businesses, public spaces, and administrative offices. For analytical purposes, we grouped these measures into two categories: adaptations to health and social service provision, and closures of businesses, administrative offices, schools, and public spaces. A total of 37 negative unintended consequences were reported in association with operational measures, the majority of which stemmed from changes in health and social services. Several studies described altered healthcare accessibility and utilisation [25, 29–31, 52], resulting from complete services closures [25, 30, 35, 57], reduced personnel [31], shortened operating hours [31], or transitions to online and telehealth services [29, 42]. One study reported that frequent changes in the availability of health services due to pandemic management protocols generated uncertainty regarding service availability, which in turn led to lower utilisation, even when services remained available in principle [29]. (Indirect) Health consequences of reduced service utilisation included higher mental health burden, increased risky sexual behaviours, and greater substance use [12, 30, 35, 56, 57]. Several studies also described non-health-related effects of adaptations to health and social service provision. For example, in crowded living conditions, telehealth services violated privacy, and cut-backs in financial and in-kind assistance from aid organisations’ exacerbated resource shortages, such as food supply [25, 29, 30, 32, 42, 47, 48, 52, 54].

The second group of operational public health and social measures–closures of businesses, administration, schools, and public spaces–caused unintended consequences at multiple levels. Business closures caused a direct loss of livelihoods, pushing migrants further into precarity, while changes to administrative operations substantially reduced or completely halted the processing of asylum claims. Studies reported direct effects on social and legal systems, which faced substantial delays and/or were unable to accept new claims [25, 30, 32, 48], as well as indirect effects on the integration of refugees and asylum seekers and on their psychosocial health and wellbeing [30, 42]. The closure of schools had direct effects on the education of camp residents. Concurrent closures of public spaces further impaired access to the internet, which is typically unavailable within camps [29, 42]. This limited access disrupted refugees’ social networks and their ability to obtain reliable information, a mechanism that negatively affected trust in pandemic measures and public authorities. Additionally, one study reported that school closures altered daily practices and behaviours of the camp residents, leading to riskier behaviours and delinquency [35]. These changes subsequently contributed to increased sexual violence, higher risk of sexually transmitted infections, and unwanted pregnancies [35].

### Nature of Unintended Consequences

While the pathway analysis primarily focused on unintended consequences with negative effects, we also identified reports of unintended consequences of a different nature. For example, Akhtar et al. reported that a lockdown in the Azraq refugee camp in Jordan was associated with a decrease in symptom severity among residents with post-traumatic stress disorder [40]. Another study described positive unintended outcomes among Rohingya refugees in Bangladesh, where supervision visits implemented for quality control of public health and social measures and mandatory testing of visitors and staff entering healthcare facilities increased trust in authorities and healthcare providers within the community [28]. In addition, Rohingya refugee women living in Cox’s Bazar, Bangladesh, identified new income-generating opportunities through the production of producing face masks, driven by the mandatory mask-wearing requirements as part of individual-level public health and social measures and shortages of available PPE [29]. Finally, at a detention centre in California, several measures–including suspension of visitations, overcrowding of medical wards, and the imposition of mass quarantine in specific units–let to a hunger strike among detainees [41]. While the value of this unintended consequence is debatable, it illustrates that detainees were able to politically organise despite differences in language, culture, and nationality, as well as the threat of penalties.

## Discussion

In this study, we explored the unintended consequences of public health and social measures implemented during the COVID-19 pandemic on refugees, asylum seekers, and IDPs living in camps and camp-like settings. The public health and social measures identified included surveillance and response measures, social and physical distancing measures, and operational measures, such as closure or changes to schools, health and social services, businesses, and public spaces. Across the included studies, we identified 113 unintended consequences affecting residents of camps and camp-like settings, covering outcomes related to health, health system functioning, economic resources, human rights, and social and institutional factors. Health-related consequences–particularly impacts on mental health and access to healthcare–were the most frequent, followed by economic effects and changes in social interactions. Overall, our findings highlight the complex interplay between public health and social measures and unintended consequences: all identified categories of measures were associated with direct and indirect negative effects on health outcomes, primarily mental health and infection risk, as well as on socio-economic conditions, including job and income loss, food insecurity, and precarious working conditions.

Despite the predominance of negative unintended consequences reported in the existing literature, some positive unintended consequences–including greater trust in the authorities and new economic opportunities–have also been described. Given that many public health and social measures amplified mistrust, and thus, contributed to negative health-related outcomes such as increased discrimination, stigmatisation, and reduced utilisation of health services, it is important to examine how, in other cases, public health and social measures unintendedly built trust; for example, through follow-up on the implementation of measures in a collaborative and non-fear-inducing manner [28]. Leveraging such positive effects can help strengthen trust in health services and authorities, both in preparation for and in response to future pandemics and health emergencies.

### Positioning of Findings Within the Literature

Our results extend the existing literature on public health and social measures in two important ways. First, while studies in the general population, specific settings or populations in vulnerable situations often focus on single measures or single outcome domains [6–9, 58] – most commonly mental health or access to services–we show that public health and social measures in camps and camp-like settings lead to interconnected and cascading unintended consequences [35, 53] across multiple domains, including health, economic stability, human rights, and social and institutional factors. Second, unlike previous reviews [58] that examined specific public health and social measures or specific population groups, our study considers all public health and social measures and explicitly traces the pathways and mechanisms through which these measures result in unintended consequences in highly constrained living environments such as camps and camp-like settings.

### Added Value of Used Frameworks

By applying the CONSEQUENT framework [5], we were able to move beyond a descriptive listing of unintended consequences and systematically identify the mechanisms and pathways through which public health and social measures affect residents of camps and camp-like settings. As mentioned previously, we observed that trust played a central role in shaping pathways and mechanisms, highlighting its crucial role as a foundation of health systems [59], particularly during health emergencies. The framework identifies five “root mechanisms” that should be considered when planning and implementing public health and social measures. These mechanisms call for counteracting actions: taking context into account, ensuring stakeholder buy-in and participation, acting on reliable evidence, addressing root causes, and strategically allocating scarce resources [5].

Using Jabeen’s framework [4] to classify unintended consequences proved challenging, as relevant information in primary studies were often unavailable, and in most cases, the unintended consequences were not clearly described, appearing instead as incidental findings within the result sections. Applying the CONSEQUENT framework as an analytical lens to identify unintended consequences and underlying mechanisms provided valuable insights into the ways public health and social measures impacted residents of camps and camp-like settings. The framework enabled a clear distinction between direct and indirect effects of measures and helped classify the mechanisms linking public health and social measures to unintended consequences. We found that the most impactful mechanisms were those that restricted refugees’ agency and self-efficacy. Understanding and considering these mechanisms and pathways–including restricted agency and self-efficacy of camp residents, mistrust, and contextual constraints–can help explain how public health and social measures lead to unintended consequences in camps and camp-like settings. This knowledge can guide the development and planning of countermeasures, ensuring that negative unintended consequences will be avoided from the outset or addressed promptly if they emerge. Public health and social measures are complex interventions implemented within complex systems and therefore require appropriate, non-linear solutions and careful planning with the engagement of all relevant stakeholders. Simple, one-size-fits-all approaches are insufficient, particularly when multiple forms of marginalisation intersect.

### Challenges and Limitations

We encountered several challenges in applying existing frameworks to our study. For example, public health and social measures were often not reported in detail, and national or regional lockdown measures, in particular, were rarely specified, which may affect the comparability and generalisability across studies. While some studies described public health and social measures–such as the detention of infected individuals or measures of spatial separation in crowded accommodations–their unintended consequences were often not analysed or supported with data. In most cases, unintended consequences were only reported “incidentally” in the discussion sections of primary studies. Only thirteen out of 35 included studies explicitly aimed to analyse unintended consequences [29, 30, 34, 35, 38, 41–43, 47, 49, 52, 53, 57], and none applied a clear concept or an *a priori* definition of unintended consequences. Future research should prioritise transparent reporting and systematic evaluation of public health and social measures to strengthen the evidence base for decision-making and to better anticipate unintended consequences, fostering more effective and equitable public health responses in refugee reception and accommodation centres.

The heterogeneity of studies conducted in camps and camp-like settings for refugees, asylum seekers, and IDPs across both high-income and low-income countries limited their direct comparability. Nevertheless, despite these differences, the settings–regardless of the country of study–share common contextual factors such as overcrowding and limited access to social and material resources, which shape living conditions. In addition, camps are often located in remote areas, restricting access to essential services such as healthcare and education. Accordingly, we assume that the outcomes and mechanisms identified in this study are likely to be similar across collective (refugee) accommodations in different countries, as well as in other institutionalised settings that limit the residents’ agency.

## Conclusion

By combining a comprehensive assessment of public health and social measures with a conceptual analysis based on existing frameworks, this study advances current understanding of how pandemic measures affect displaced populations living in camps and camp-like settings. Overall, there is substantial evidence of unintended consequences of public health and social measures–in particular social and physical distancing measures, such as lockdowns–that negatively impact the mental health of refugees, asylum seekers, and IDPs in these settings, both directly and indirectly. These unintended consequences are closely linked to interruptions of social support and networks, precarious economic conditions, and increases in risky behaviours, such as substance use. Limited access to health services further exacerbates these impacts. Negative consequences were observed across both high- and low-income countries. Nevertheless, the evidence remains incomplete, as unintended consequences of public health and social measures were often not the primary focus of studies and were frequently reported only incidentally.

From a practical perspective, it is essential to systematically consider unintended consequence of public health and social measures in emergency preparedness plans and to develop and implement pre-emptive measures to mitigate them, particularly in camps and camp-like settings such as reception and collective accommodation centres for refugees. Given that mistrust and uncertainty emerged as key mechanisms driving negative unintended consequences, public health and social measures must incorporate trust-building strategies into health interventions both before and during emergencies. Trust and trust-building should be integral to the design of public health and social measures, rather than optional components considered only during implementation. Future planning of control measures should explicitly account for root mechanisms and involve all relevant stakeholders across disciplines to ensure interventions are effective, equitable, and context-sensitive.
