# Supplementary material for: Unintended Consequences of COVID-19 Public Health and Social Measures in Camps and Camp-Like Settings: A Systematic Review and Conceptual Analysis
Source: Public Health Rev. 2026 Feb 25;47:1608732. doi: 10.3389/phrs.2026.1608732 (PMC12976605; doi:10.3389/phrs.2026.1608732)
Supplement: Supplementary file 1 [file DataSheet1.pdf]

Supplement

Unintended Consequences of COVID-19 Public Health and Social Measures in Camps and Camp-like Settings: A Systematic Review and Conceptual Analysis

Content

1. PECO Scheme 2

Table S1: PECO Scheme 2

2. Search Strings 2

Table S2a: WHO COVID-19 database: 17.03.2023 2

Table S2b: The Cochrane Library: 17.03.2023 3

3. Inclusion/Exclusion criteria 4

Table S3: Inclusion and Exclusion criteria 4

4. Methodology mosaic plots 5

Figure S1a: Proportion of unintended consequences by pandemic control measures (PHSM) with broad (left) and granular aggregation (right) (weighted by quality of studies) 6

Figure S1b: Proportion of unintended consequences by pandemic control measures (PHSM) with broad (left) and granular aggregation (right) (weighted by focus of the analysis) 6

5. Quality Appraisal 7

Table S4a: Quality Appraisal of Cross-Sectional Studies 7

Table S4b: Quality Appraisal of Qualitative Studies 8

Table S4c: Quality Appraisal of Cohort Studies 9

Table S4d: Quality Appraisal of Quasi Experimental Studies 10

Table S4e: Quality Appraisal of Systematic review 10

Table S4f: Quality Appraisal of Randomised controlled trials 10

6. Cascading diagrams for PHSM 11

Figure S2a: Pathways and mechanisms towards unintended consequences stemming from surveillance and response measures in specific settings 11

Figure S2b: Pathways and mechanisms towards unintended consequences stemming from social and physical distancing measures 12

Figure S2c: Pathways and mechanisms towards unintended consequences stemming from operational measures 13

7. Cascading diagrams for PHSM 14

Table S5: PRISMA Checklist for Abstracts 14

Table S6: PRISMA Checklist (Main Article) 15

1. PECO Scheme

Table S1: PECO Scheme

	Term	Definition
**P**opulation	Refugees, asylum seekers and IDP in camps, reception centres and collective accommodation centres	Refugees:<br>Refugee definition of 1951 Refugee Convention: “A refugee, according to the Convention, is someone who is unable or unwilling to return to their country of origin owing to a well-founded fear of being persecuted for reasons of race, religion, nationality, membership of a particular social group, or political opinion.” (UNHCR, 2010)<br><br>We further consider refugees as umbrella term for people seeking international protection but whose application has not yet been processed, is ongoing, or has been rejected as asylum seekers.<br><br>Asylum seekers:<br>An individual who is seeking international protection. In countries with individualized procedures, an asylum seeker is someone whose claim has not yet been finally decided on by the country in which he or she has submitted it. Not every asylum seeker will ultimately be recognized as a refugee, but every recognized refugee is initially an asylum seeker. (IOM, 2019).<br><br>IDP:<br>Persons or groups of persons who have been forced or obliged to flee or to leave their homes or places of habitual residence, in particular as a result of or in order to avoid the effects of armed conflict, situations of generalized violence, violations of human rights or natural or human-made disasters, and who have not crossed an internationally recognized State border (IOM, 2019).
**E**xposure	Public Health and social measures	Exposure to policy measures, policies/action plans and/or outbreak prevention and control measures implemented in camps, reception centres, and collective accommodation centres for refugees, asylum seekers, and IDP to prevent and reduce SARS-CoV-2 infections and their consequences. This includes PHSMs that have also been implemented outside the defined setting, but are specifically used within the setting to protect the specified population. They may include, but are not restricted to: individual, environmental, preparedness and response, social and physical distancing, international travel, drug-based or biological classes of measures.
**C**omparison	None/general population	Either no comparison group or comparison with other population groups (migrant populations, or general population), including other institutionalized settings. Comparison groups will not be used as inclusion criteria.
**O**utcome	Any domain of health, health care, and social and economic outcomes	Broadly, all studies empirically assessing consequences of PHSMs implemented in camps, reception centres, or collective accommodation centres are included.<br><br>**Specific outcomes are:**<br>• Any domain of health (mental, physical etc.)<br>• Healthcare (e.g. healthcare access)<br>• Social and economic consequences that may directly or indirectly affect health (e.g. job loss etc.) (Stratil et al., 2020)

2. Search Strings

Table S2a: WHO COVID-19 database: 17.03.2023

ID	Query	Results
#6	#3 AND #4 AND #5	1.710
#5	(da:((202112$ OR 2022$ OR 202301 OR 202302)))	307540
#4	(tw:(camp* OR accomoda* OR ((reception OR accommodation OR detention) AND (centr* OR center* OR facility OR facilities OR placement* OR house*)) OR reception* OR centre* OR	102372

	center* OR shelter* OR "camplike setting" OR detention OR placement* OR housing* OR house* or "refugee camp*"))	
#3	#1 AND NOT #2	17614
#2	(tw:((migraine* OR (migra* AND (cell* OR metastas* OR inflamma* OR diffusion* OR catheter* OR tumor OR cancer)) OR extracellular* OR intracellular*) OR (transient* AND (accumulat* OR receptor OR focal OR cerebral OR ischaemic OR response OR absorption OR suppression OR hypocalcemia OR gene OR transgene OR tube))))	6482
#1	(tw:(refugee OR "asylum seeker" OR asyl* OR (displaced AND (person* OR people)) OR migra* OR (forced AND migra*) OR migration OR immigra* OR "human migration" OR stateless OR "state-less" OR (irregular AND migra*) OR (undocumented AND migra*) OR "internally displaced" OR detainees OR "residence status" OR "foreign-born" OR "displaced person" OR noncitizen OR outsider OR newcomer OR "newly arrived" OR "new arrival" OR "recent entrant" OR "non national" OR "non-national" OR transient OR minoritie* OR ethnic OR "Transients and Migrants"))	20556

Table S2b: The Cochrane Library: 17.03.2023

ID	Query	Results
**#17**	**#16 publication limit 01.12.2021 to 28.02.2023**	**35**
**#16**	**#6 AND #12 AND #15**	**70**
#15	#13 OR #14	200196
#14	(camp*:ti,ab,kw OR accomoda*:ti,ab,kw OR ((reception:ti,ab,kw OR accommodation:ti,ab,kw OR detention:ti,ab,kw) AND (centr*:ti,ab,kw OR center*:ti,ab,kw OR facility:ti,ab,kw OR facilities:ti,ab,kw OR placement*:ti,ab,kw OR house*:ti,ab,kw)) OR reception*:ti,ab,kw OR centre*:ti,ab,kw OR center*:ti,ab,kw OR shelter*:ti,ab,kw OR "camplike setting":ti,ab,kw OR detention:ti,ab,kw OR placement*:ti,ab,kw OR housing*:ti,ab,kw OR house*:ti,ab,kw)	200196
#13	MeSH descriptor: [Refugee Camps] explode all trees	12
#12	(#7 OR #8 OR #9 OR #10) NOT #11	17603
#11	((migraine*:ti,ab,kw OR (migra*:ti,ab,kw AND (cell*:ti,ab,kw OR metastas*:ti,ab,kw OR inflamma*:ti,ab,kw OR diffusion*:ti,ab,kw OR catheter*:ti,ab,kw OR tumor:ti,ab,kw OR cancer:ti,ab,kw)) OR extracellular*:ti,ab,kw OR intracellular*:ti,ab,kw) OR (transient*:ti,ab,kw AND (accumulat*:ti,ab,kw OR receptor:ti,ab,kw OR focal:ti,ab,kw OR cerebral:ti,ab,kw OR ischaemic:ti,ab,kw OR response:ti,ab,kw OR absorption:ti,ab,kw OR suppression:ti,ab,kw OR hypocalcemia:ti,ab,kw OR gene:ti,ab,kw OR transgene:ti,ab,kw OR tube:ti,ab,kw)))	28920
#10	refugee:ti,ab,kw OR “asylum seeker”:ti,ab,kw OR “asyl*”:ti,ab,kw OR (displaced:ti,ab,kw AND (person*:ti,ab,kw OR people:ti,ab,kw)) OR “migra*”:ti,ab,kw OR “forced migra*”:ti,ab,kw OR migration:ti,ab,kw OR immigra*:ti,ab,kw OR “human migration”:ti,ab,kw OR stateless:ti,ab,kw OR “state-less”:ti,ab,kw OR “irregular migra*”:ti,ab,kw OR “regular migra*”:ti,ab,kw OR “undocumented migra*”:ti,ab,kw OR “internally displaced”:ti,ab,kw OR “detainees”:ti,ab,kw OR “residence status”:ti,ab,kw OR “foreign-born”:ti,ab,kw OR “ displaced person”:ti,ab,kw OR “noncitizen”:ti,ab,kw OR “outsider”:ti,ab,kw OR “newcomer”:ti,ab,kw OR “newly arrived”:ti,ab,kw OR “new arrival”:ti,ab,kw OR “recent entrant”:ti,ab,kw OR “non national”:ti,ab,kw OR “non-national”:ti,ab,kw OR “transient”:ti,ab,kw OR “minorities”:ti,ab,kw OR “ethnic”:ti,ab,kw	29699
#9	MeSH descriptor: [Refugees] explode all trees	228
#8	MeSH descriptor: [Human Migration] explode all trees	79
#7	MeSH descriptor: [Transients and Migrants] explode all trees	104
#6	#1 OR #2 OR #3 OR #4 OR #5	15142
#5	("corona virus":ti,ab,kw OR "corona viruses":ti,ab,kw OR coronavir*:ti,ab,kw OR coronovirus*:ti,ab,kw OR betacoronavirus*:ti,ab,kw) AND (novel:ti,ab,kw OR 2019:ti,ab,kw OR Wuhan:ti,ab,kw OR Huanan:ti,ab,kw OR Hubei:ti,ab,kw) OR "new coronavirus":ti,ab,kw OR "COVID-19":ti,ab,kw OR COVID19:ti,ab,kw OR "SARS coronavirus 2":ti,ab,kw OR "severe acute respiratory syndrome coronavirus 2":ti,ab,kw OR nCoV:ti,ab,kw OR 2019nCoV:ti,ab,kw OR nCoV2019:ti,ab,kw OR "SARS-CoV-2":ti,ab,kw OR "SARS-CoV2":ti,ab,kw OR SARSCoV19:ti,ab,kw OR SARS-CoV19:ti,ab,kw OR SARS-CoV-19:ti,ab,kw OR HCoV-19:ti,ab,kw OR WN-CoV:ti,ab,kw	15063
#4	MeSH descriptor: [SARS-CoV-2] explode all trees	2174
#3	MeSH descriptor: [COVID-19] explode all trees	3974
#2	MeSH descriptor: [Coronavirus] explode all trees	2204
#1	MeSH descriptor: [Coronavirus Infections] explode all trees	4555

3. Inclusion/Exclusion criteria

Table S3: Inclusion and Exclusion criteria

**Inclusion/Exclusion criteria**		
**Criteria**	**Inclusion**	**Exclusion**
**Type of Population**	Refugees, asylum seekers, and IDP living in camps, reception centres, and collective accommodation centres.	Unclear populations.
**Type of studies**	• Empirical quantitative studies (cross-sectional, case-control, cohort, intervention studies, ecological studies, modelling studies, epidemiological outbreak reports).<br>• Empirical qualitative studies (interviews, focus groups, ethnography) analysing health outcomes or impact of lock-down measures and evaluation strategies that include refugees, asylum seekers and IDP living in camps, reception centres, and collective accommodation centres.<br>• Mixed-methods studies where qualitative and quantitative parts where reported separately.<br>• Identified rapid or systematic reviews will only be screened for their included articles; meta-analyses will be included on their own.<br>• Policy analysis with empirical data.	Case series, theoretical research work; Policy analysis without empirical data.
**Type of articles**	Published peer-reviewed articles and preprints identified, as well as official reports from IOM, ECDC, or EUPHA or other pertinent websites.	Grey literature except for official reports on mentioned websites; Book sections; Commentaries and Letters to the Editor, unless reporting results of a study in a transparent scientific structure (IMRaD). Pure opinion pieces that draw upon data (without transparent description of methods) will be excluded.
**Focus of study**	• Studies reporting consequences of PHSMs among refugees, asylum seekers, or IDP living in camps, reception centres or collective accommodation centres.<br>• For full-text stage: studies reporting PHSM among refugees, asylum seekers, or IDP living in camps, reception centres, or collective accommodation centres.	Studies with no focus on consequences of PHSMs among refugees, asylum seekers, or IDP living in camps, reception centres or collective accommodation centres.
**Type of outcome measure**	• Any health-related outcomes<br>• Healthcare-related outcomes<br>• Social and economic consequences that may directly or indirectly affect health (job loss, income loss, etc.)	No health- or healthcare-related outcomes; and/or no health-related social and economic consequences.
**Geographical area**	Worldwide	No studies will be excluded based on geography.
**Language of publication**	Studies published in English, German or Spanish will be included (but searches will be conducted only in English).	Studies in other languages than English, German or Spanish.
**Date of Publication**	Studies published since December 2019.	

4. Methodology mosaic plots

We used mosaic plots to visualise the proportion of each unintended consequence, stratified by pandemic control measures, at two levels of aggregation. The unintended consequences were categorised into the following groups based on the CONSEQUENT framework[1]: 'Acceptability and compliance', 'Environmental', 'Economic and resources', 'Health system', 'Human and fundamental rights', 'Mental health', 'Physical health', and 'Social and/or institutional'.

At the first level of aggregation, pandemic control measures were grouped according to the WHO taxonomy (REF) as 'individual measures', 'social and physical distancing measures', 'surveillance and response measures', and 'other'. At the second level of aggregation, control measures were categorised into more specific actions: 'wearing a mask', 'using hand hygiene', 'using other personal protective equipment', 'domestic travel', 'international travel measures', 'offices, businesses, institutions, and operations', 'gatherings, businesses, and services', 'school measures', 'case finding and isolation', and 'special populations'.

The plots were developed using programming language R 4.3.2[2] within the integrated development environment R Studio 2023.06.0 421. We decided to use unweighted plots after conducting a sensitivity analysis, applying weights to assess the impact of the quality of studies and their focus on unintended consequences. Weights were based on two factors: the quality of the studies (low=1, medium=2, high=3) and whether the unintended consequences were the primary focus of the analysis (no=1, yes=2). These weights were applied to each unintended consequence of each pandemic control measure for every included study. In a scenario were two categories of unintended consequences had the same frequency, categories with higher weights across all studies took up a larger portion of the mosaic plot compared to those with lower overall weights. This means the weights help determine whether factors like study quality or the focus of the study's analysis on unintended consequences affect the importance of certain categories of unintended consequences. Since, the weights are quite evenly distributed across unintended consequences, they only slightly alter the proportion of the mosaic plots. As a result, the weighted and unweighted graphs appear quite similar. We chose to use the unweighted graphs in the mauscript because they offer a more objective representation of the data, without the potential influence of subjective weighting factors.

[1] Stratil JM, Biallas RL, Movsisyan A, Oliver K, Rehfuess EA. Development of an overarching framework for anticipating and assessing adverse and other unintended consequences of public health interventions (CONSEQUENT): a best-fit framework synthesis. BMJ Public Health. 2024;2(1):e000209.

[2] https://www.R-project.org/, within the integrated development environment R Studio 2023.06.0 421: (http://www.posit.co/.

Figure S1a: Proportion of unintended consequences by pandemic control measures (PHSM) with broad (left) and granular aggregation (right) (weighted by quality of studies)

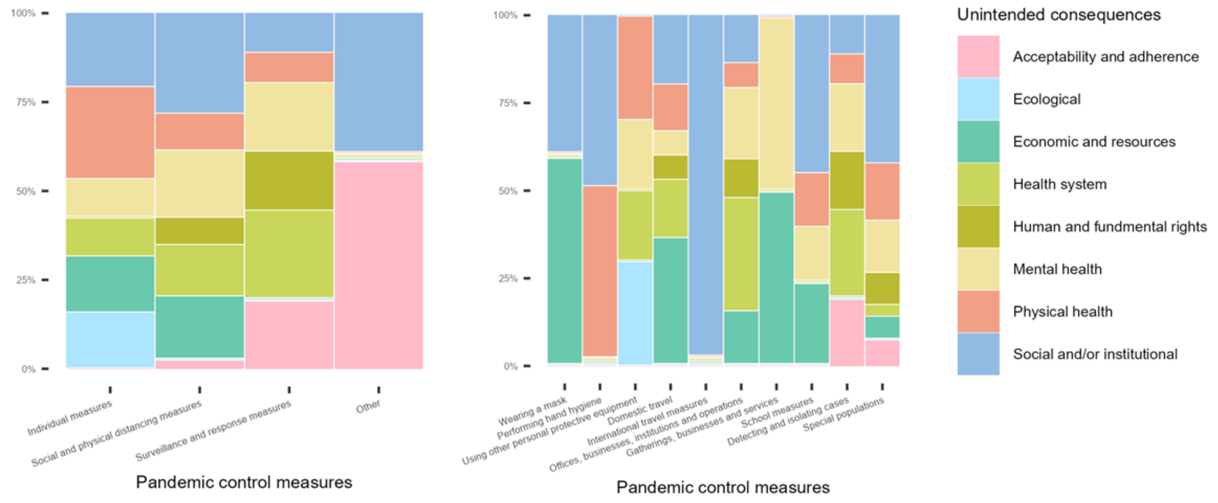

S1a) Proportions with broad PHSM categories weighted by quality of studies

S1a) Proportions with granular PHSM categories weighted by quality of studies

Figure S1b: Proportion of unintended consequences by pandemic control measures (PHSM) with broad (left) and granular aggregation (right) (weighted by focus of the analysis)

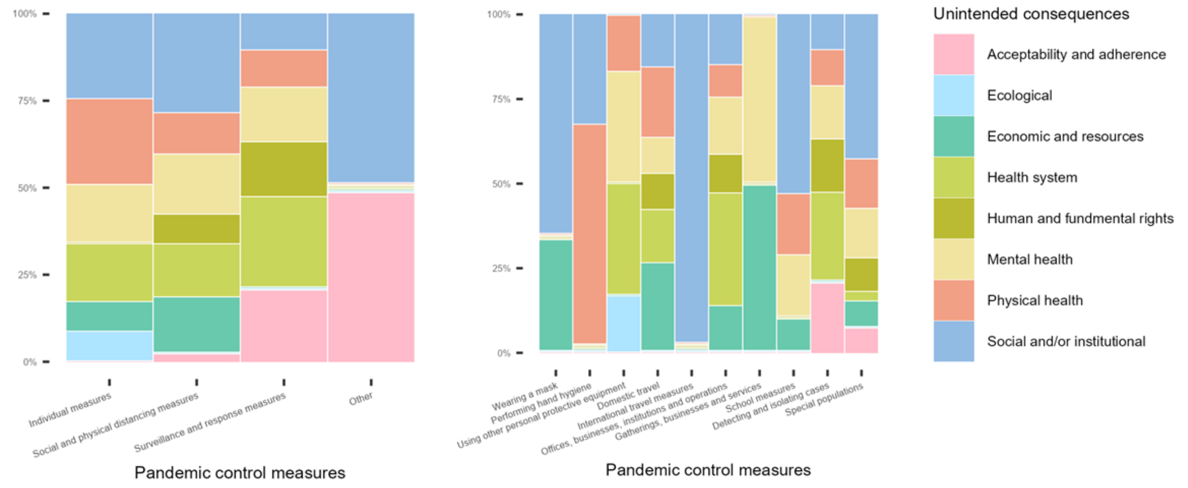

S1b) Proportions with broad PHSM categories weighted by focus of the analysis

S1b) Proportions with granular PHSM categories weighted by focus of the analysis

5. Quality Appraisal

Tables S4a-f show the quality appraisal for each study by group of study design based on the JBI checklists.

Table S4a: Quality Appraisal of Cross-Sectional Studies

Author & Year	1. Clear inclusion criteria		2. Detailed description of subjects and setting		3. Valid and reliable mesaure of Exposure(s)		4. Objective measure of condition(s)		5. Confounder identified		6. Confounder addressed		7. Valid and reliable measure of Outcome(s)		8. Appropriate statistical analysis		Overall Rating		Final Rating	
	R1	R2	R1	R2	R1	R2	R1	R2	R1	R2	R1	R2	R1	R2	R1	R2	R1	R2	Mean Score	Cat.
Abu Hamad et al. 2022*	yes	yes	yes	yes	unclear	na	yes	na	yes	no	no	no	yes	yes	unclear	unclear	75.0	58.0	67.0	medium
Altare et al. 2022	yes	yes	yes	yes	yes	yes	yes	yes	unclear	yes	unclear	yes	yes	yes	yes	yes	88.0	100.0	94.0	high
Hamdan et al. 2021	yes	na	na	yes	na	na	unclear	na	yes	yes	yes	yes	no	yes	no	yes	50.0	100.0	75.0	high
Kondilis et al. 2021	yes	yes	yes	yes	yes	na	yes	na	unclear	no	unclear	na	yes	yes	yes	yes	75.0	80.0	77.5	high
Korobkova et al. 2022	no	no	no	no	na	unclear	na	yes	na	na	no	na	unclear	unclear	yes	unclear	30.0	42.0	36.0	low
Mistry et al. 2021	yes	yes	yes	yes	na	no	na	na	yes	no	yes	no	yes	yes	yes	yes	100.0	57.0	79.0	high
Mumin et al. 2022	yes	yes	yes	no	yes	na	na	na	yes	yes	yes	yes	unlcear	yes	yes	yes	93.0	83.0	88.0	high
Stillman et al. 2022	yes	yes	yes	yes	yes	na	na	yes	no	yes	no	yes	yes	yes	no	yes	57.0	100.0	79.0	high

*mixed method study; na = not applicable

Table S4b: Quality Appraisal of Qualitative Studies

Author & Year	1. Congruity between the stated philosophical perspective and the research methodology		2. Congruity between the research methodology and the research question or objectives		3. Congruity between the research methodology and the methods used to collect data		4. Congruity between the research methodology and the representation and analysis of data		5. Congruity between the research methodology and the interpretation of results		6. Trancparent culturally or theoretically position stated		7. Influence of the researcher on the research, and vice- versa, addressed		8. Participants adequately represented		9. Evidence of ethical approval and ethical methodology		10. Conclusions drawn flow from the analysis, or interpretation, of the data		Overall Rating		Final Rating	
	R1	R2	R1	R2	R1	R2	R1	R2	R1	R2	R1	R2	R1	R2	R1	R2	R1	R2	R1	R2	R1	R2	Mean Score	Cat.
Abu Hamad et al. 2022*	yes	unclear	yes	unclear	yes	yes	yes	yes	yes	unclear	no	no	no	no	yes	yes	yes	yes	yes	yes	80.0	65.0	73.0	medium
Ag Ahmed et al. 2021	unclear	yes	yes	yes	na	yes	yes	yes	yes	yes	no	unclear	no	no	yes	yes	yes	yes	yes	yes	72.0	85.0	79.0	high
Apolot et al. 2023	yes	yes	yes	yes	yes	yes	yes	yes	yes	yes	no	no	no	no	yes	yes	yes	na	yes	yes	80.0	78.0	79.0	high
Asoni et al. 2023	yes	yes	yes	yes	yes	yes	unclear	yes	unclear	yes	yes	yes	yes	no	unclear	yes	na	yes	yes	yes	83.0	90.0	87.0	high
Avalos Cortez & van Blerk 2021	no	no	yes	unclear	unclear	unclear	yes	unclear	yes	no	no	no	no	no	yes	yes	no	no	yes	unclear	50.0	10.0	30.0	low
Berg 2022	no	yes	yes	yes	yes	yes	yes	yes	yes	yes	no	no	no	no	yes	yes	no	unclear	yes	yes	60.0	75.0	68.0	medium
Berg et al. 2022	yes	yes	yes	yes	yes	yes	yes	yes	yes	yes	no	yes	no	yes	yes	yes	yes	yes	yes	yes	80.0	100.0	90.0	high
Boehme et al. 2022	yes	yes	yes	yes	yes	yes	yes	yes	yes	yes	no	unclear	no	no	no	yes	no	unclear	yes	yes	60.0	80.0	70.0	medium
Can Collado et al. 2021	no	yes	yes	yes	unclear	unclear	unclear	unclear	unclear	yes	no	no	no	no	yes	yes	yes	yes	yes	yes	55.0	70.0	63.0	medium
Chowdhury et al. 2022	yes	yes	yes	yes	yes	yes	yes	yes	yes	yes	yes	yes	yes	yes	yes	yes	yes	yes	yes	yes	100.0	100.0	100.0	high
Crouzet et al. 2022	yes	unclear	yes	unclear	yes	yes	yes	yes	yes	yes	no	unclear	no	unclear	yes	yes	yes	yes	yes	yes	80.0	80.0	80.0	high
Cruz Pineiro & Ibarra	yes	yes	yes	yes	yes	yes	yes	yes	yes	yes	no	unclear	no	unclear	yes	yes	yes	yes	yes	yes	80.0	90.0	85.0	high

2022																								
Da Mosto et al. 2021	yes	no	yes	no	yes	yes	yes	yes	yes	yes	no	no	unclear	no	yes	yes	yes	yes	yes	yes	80.0	60.0	70.0	medium
Filippi & Giliberti 2021	no	no	yes	yes	yes	yes	yes	yes	yes	yes	no	no	no	no	yes	yes	no	no	yes	yes	60.0	60.0	60.0	medium
Filosi et al. 2022	yes	yes	unclear	yes	yes	yes	unclear	yes	unclear	yes	no	no	no	no	yes	yes	unclear	unclear	yes	yes	60.0	75.0	68.0	medium
Ghaddar et al. 2023	yes	unclear	yes	yes	yes	yes	yes	yes	yes	yes	no	no	no	no	yes	yes	yes	yes	yes	yes	80.0	75.0	78.0	high
Infante et al. 2022	no	no	yes	yes	yes	yes	yes	yes	no	yes	no	no	no	no	yes	yes	yes	yes	yes	no	60.0	60.0	60.0	medium
Meyer et al. 2022	no	no	yes	yes	yes	unclear	yes	yes	yes	no	no	no	no	no	yes	yes	yes	yes	yes	no	70.0	45.0	58.0	medium
Ozer et al. 2022	no	unclear	yes	yes	yes	yes	yes	unclear	yes	yes	no	no	no	no	yes	yes	unclear	yes	yes	yes	65.0	70.0	68.0	medium
Rangel Gomez et al. 2023	yes	yes	yes	yes	no	yes	yes	yes	yes	yes	no	no	no	no	no	no	yes	yes	unclear	yes	55.0	70.0	63.0	medium
Reynolds et al. 2022	no	yes	yes	yes	yes	yes	yes	yes	yes	no	yes	no	no	no	yes	yes	no	yes	yes	yes	60.0	80.0	70.0	medium
Tschalaer 2022	yes	yes	yes	yes	yes	yes	yes	yes	yes	yes	yes	yes	unclear	yes	yes	yes	unclear	yes	yes	yes	90.0	100.0	95.0	high

*mixed method study; na = not applicable

Table S4c: Quality Appraisal of Cohort Studies

Author & Year	1. Two groups recruited from the same population		2. Exposure measured similar for two groups		3. Exposure measured valid reliable		4. Confounding factors identified		5. Strategies to deal with confounding factors stated		6. Groups were free of the outcome at the start of the study		7. Outcomes measured in a valid and reliable way		8. Follow up time reported and sufficient		9. Follow up complete or reasons to loss to follow up described and explored		10. Strategies to address incomplete follow up utilized		11. Appropriate statistical analysis used		Overall Rating		Final Rating	
	R1	R2	R1	R2	R1	R2	R1	R2	R1	R2	R1	R2	R1	R2	R1	R2	R1	R2	R1	R2	R1	R2	R1	R2	Mean Score	Cat
Kizilhan & Noll-Hussong 2020	na	yes	na	yes	na	no	na	yes	na	no	na	na	na	yes	na	yes	na	unclear	na	no	na	yes	0.0	60.0	30.0	low
Nwadiuko et al. 2023	na	na	yes	yes	unclear	no	yes	na	unclear	no	yes	yes	yes	yes	yes	yes	unclear	no	unclear	no	yes	yes	83.0	63.0	73.0	medium

na = not applicable

Table S4d: Quality Appraisal of Quasi Experimental Studies

Author & Year	1. Cause and Effect clearly defined		2. Was there a control group?		3. Bias related to confounding factors		4. Administration of intervention/ exposure		5. Multiple measure-ments		6. Outcomes measured in the same way		7. Outcomes measured in a reliable way		8. Follow up complete		9. Appropriate statistical analysis		Overall Rating		Final Rating	
	R1	R2	R1	R2	R1	R2	R1	R2	R1	R2	R1	R2	R1	R2	R1	R2	R1	R2	R1	R2	Mean Score	Cat
Stein et al. 2022	unclear	yes	yes	yes	yes	yes	yes	yes	yes	yes	yes	yes	yes	yes	yes	yes	yes	yes	94.0	100.0	97.0	high

Table S4e: Quality Appraisal of Systematic review

Author & Year	1. Clear Review question		2. Appro-priate inclusion criteria		3. Appropriate search strategy		4. Adequate sources and resources used		5. Appropriate critical appraisal		6. Critical appraisal independently assessed		7. Methods for data extraction		8. Approp-riate methods for synthesis		9. Publi-caiton bias assessed		10. Recom-mendations for policy/ practice		11. Appropriate directives for new research		Overall Rating		Final Rating	
	R1	R2	R1	R2	R1	R2	R1	R2	R1	R2	R1	R2	R1	R2	R1	R2	R1	R2	R1	R2	R1	R2	R1	R2	Mean Score	Cat
Jahn et al. 2022	yes	yes	yes	yes	yes	yes	yes	yes	na	na	yes	na	unclear	unclear	yes	yes	na	yes	yes	yes	yes	na	94.0	100.0	97.0	high
na = not applicable																										

Table S4f: Quality Appraisal of Randomised controlled trials

Author & Year	1. Random-isation of participants		2. Concealed group allocation		3. Similar groups at baseline		4. Blinding of participants		5. Blinding of caregivers		6. Blinding of assessors		7. Treat-ment of groups		8. Follow up complete		9. Inten-tion to treat analysis		10. Out-come measured the same		11. Reliable outcome measures		12. Appro-priate statistical methods		13. Appro-priate study design		Overall Rating		Final Rating	
	R1	R2	R1	R2	R1	R2	R1	R2	R1	R2	R1	R2	R1	R2	R1	R2	R1	R2	R1	R2	R1	R2	R1	R2	R1	R2	R1	R2	Mean Score	Cat
Akhtar et al. 2021	yes	yes	unclear	unclear	yes	no	no	yes	no	no	yes	unclear	yes	yes	no	unclear	yes	yes	yes	yes	yes	yes	yes	yes	yes	yes	73.0	73.0	73.0	medium

6. Cascading diagrams for PHSM

The figures S1, S2, and S3 show the mechanisms and pathways at play for each group of PHSM. Specific measures ar highlighted in the purple or beige boxes. Mechanisms are displayed in diamonds (different colours indicate different domains according to the CONSEQUENT framework by Stratil et al. (REF)). The arrows indicate the pathways, that can be either direct (solid line) or indirect (dashed line). The pink boxed show health and health-related unintended consequences based on the categorisation suggested in the CONSEQUENT framework.

Figure S2a: Pathways and mechanisms towards unintended consequences stemming from surveillance and response measures in specific settings

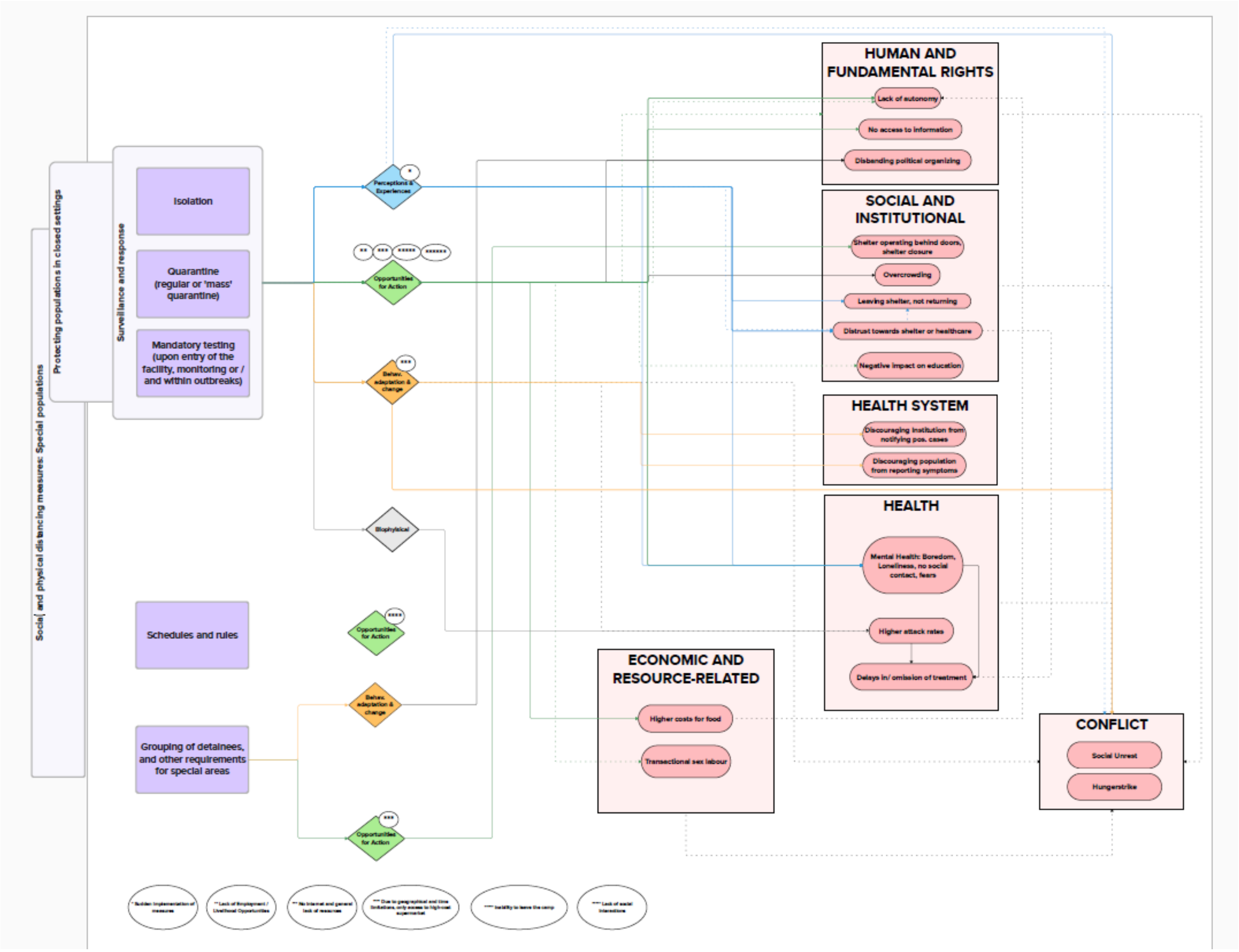

Figure S2b: Pathways and mechanisms towards unintended consequences stemming from social and physical distancing measures

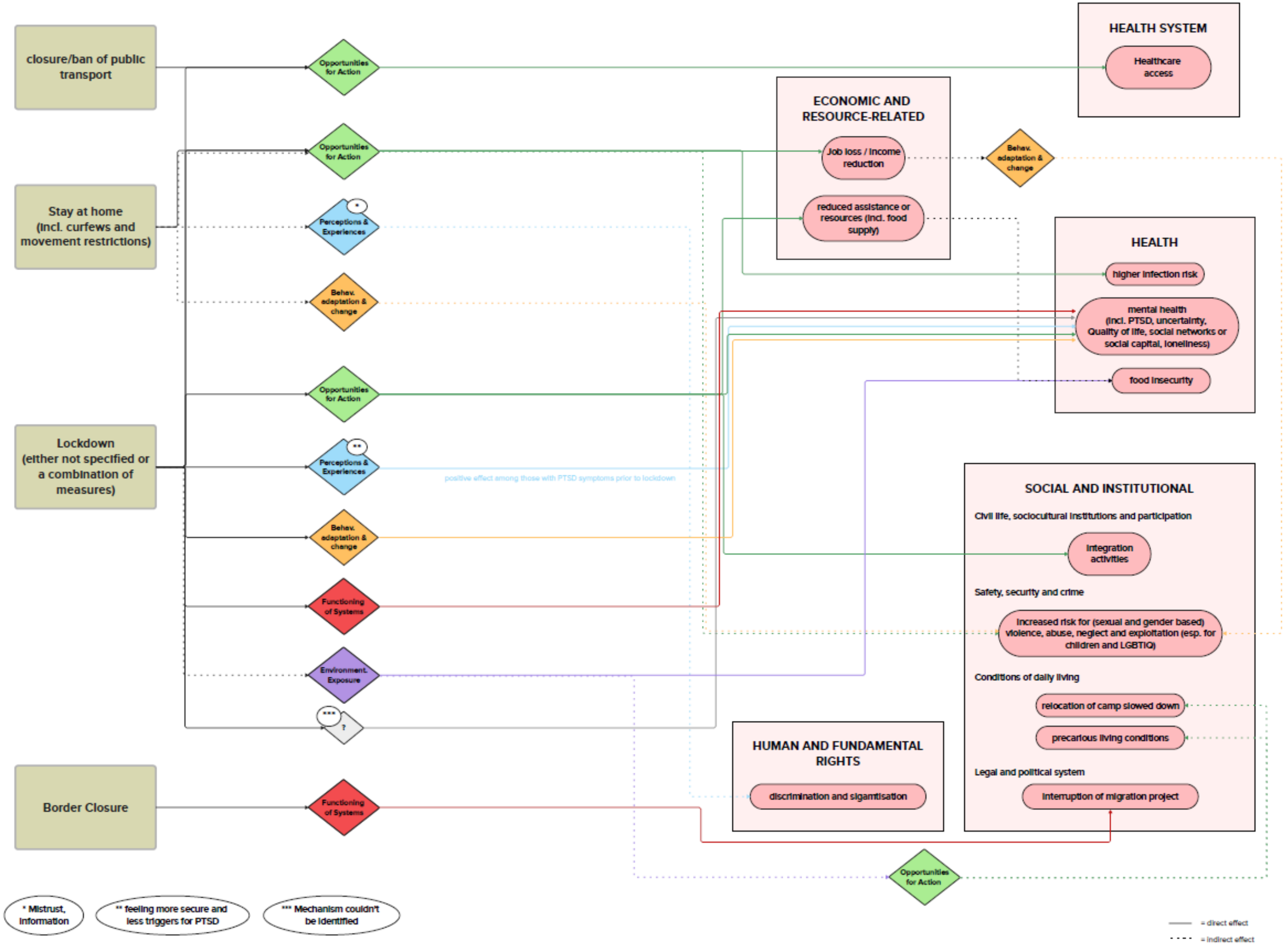

Figure S2c: Pathways and mechanisms towards unintended consequences stemming from operational measures

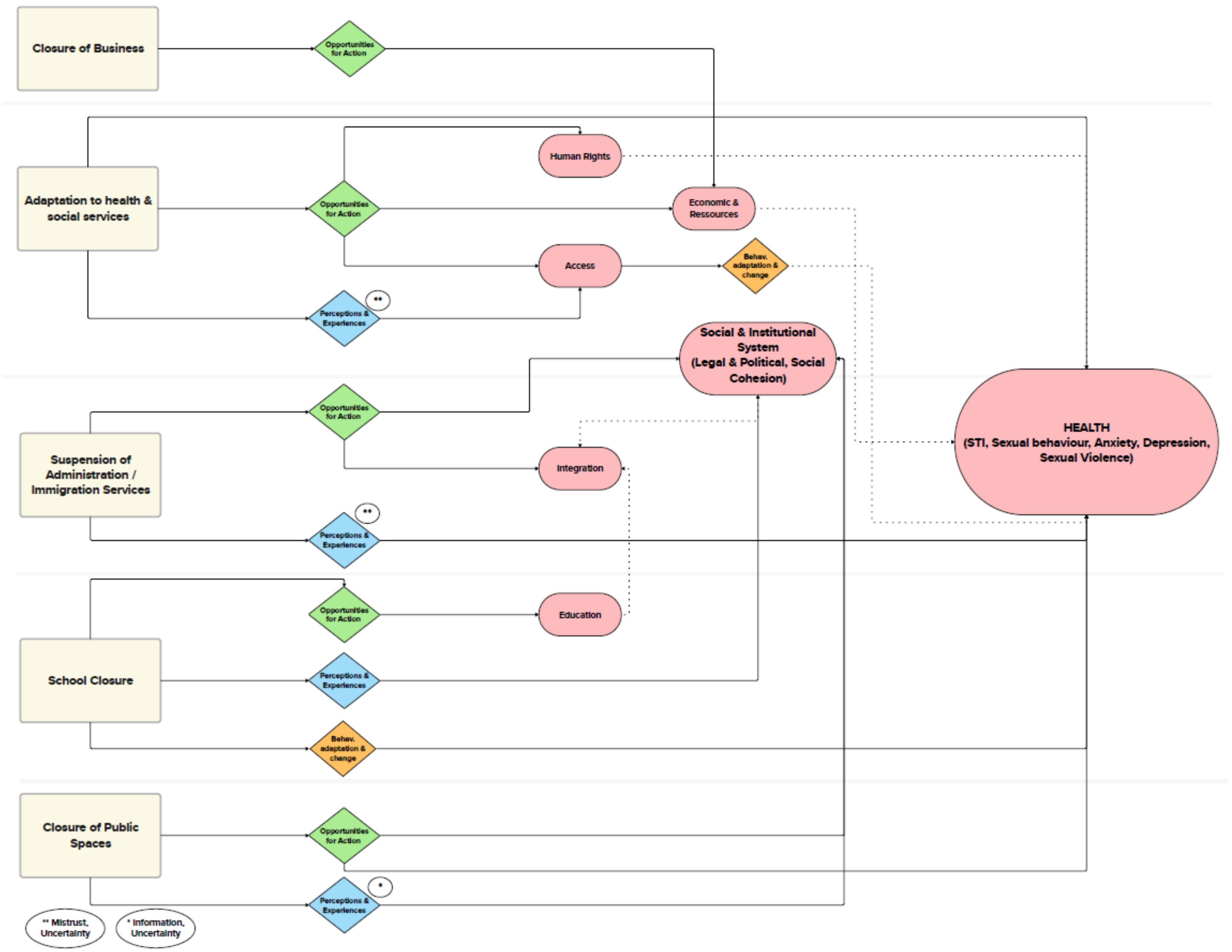

7. Cascading diagrams for PHSM

The following pages contain the PRISMA Checklists for abstract and the main article.

Table S5: PRISMA Checklist for Abstracts

Section and Topic	Item #	Checklist item	Reported (Yes/No)
**TITLE**			
Title	1	Identify the report as a systematic review.	Yes
**BACKGROUND**			
Objectives	2	Provide an explicit statement of the main objective(s) or question(s) the review addresses.	Yes
**METHODS**			
Eligibility criteria	3	Specify the inclusion and exclusion criteria for the review.	No
Information sources	4	Specify the information sources (e.g. databases, registers) used to identify studies and the date when each was last searched.	No
Risk of bias	5	Specify the methods used to assess risk of bias in the included studies.	No
Synthesis of results	6	Specify the methods used to present and synthesise results.	Yes
**RESULTS**			
Included studies	7	Give the total number of included studies and participants and summarise relevant characteristics of studies.	No
Synthesis of results	8	Present results for main outcomes, preferably indicating the number of included studies and participants for each. If meta-analysis was done, report the summary estimate and confidence/credible interval. If comparing groups, indicate the direction of the effect (i.e. which group is favoured).	Yes
**DISCUSSION**			
Limitations of evidence	9	Provide a brief summary of the limitations of the evidence included in the review (e.g. study risk of bias, inconsistency and imprecision).	No
Interpretation	10	Provide a general interpretation of the results and important implications.	Yes
**OTHER**			
Funding	11	Specify the primary source of funding for the review.	No
Registration	12	Provide the register name and registration number.	No

Table S6: PRISMA Checklist (Main Article)

Section and Topic	Item #	Checklist item	Location where item is reported
**TITLE**			
Title	1	Identify the report as a systematic review.	Title page (p.1)
**ABSTRACT**			
Abstract	2	See the PRISMA 2020 for Abstracts checklist.	Supplement Table S5
**INTRODUCTION**			
Rationale	3	Describe the rationale for the review in the context of existing knowledge.	p.2 Introduction, 1st paragraph
Objectives	4	Provide an explicit statement of the objective(s) or question(s) the review addresses.	p.2 Introduction, 2nd paragraph
**METHODS**			
Eligibility criteria	5	Specify the inclusion and exclusion criteria for the review and how studies were grouped for the syntheses.	p.3, Eligibility criteria
Information sources	6	Specify all databases, registers, websites, organisations, reference lists and other sources searched or consulted to identify studies. Specify the date when each source was last searched or consulted.	p.3, Search strategy
Search strategy	7	Present the full search strategies for all databases, registers and websites, including any filters and limits used.	Supplement, Table S1 & S2a,b
Selection process	8	Specify the methods used to decide whether a study met the inclusion criteria of the review, including how many reviewers screened each record and each report retrieved, whether they worked independently, and if applicable, details of automation tools used in the process.	p.3f, Screening process
Data collection process	9	Specify the methods used to collect data from reports, including how many reviewers collected data from each report, whether they worked independently, any processes for obtaining or confirming data from study investigators, and if applicable, details of automation tools used in the process.	p.4, Data extraction and management
Data items	10a	List and define all outcomes for which data were sought. Specify whether all results that were compatible with each outcome domain in each study were sought (e.g. for all measures, time points, analyses), and if not, the methods used to decide which results to collect.	p.4, Data extraction and management
	10b	List and define all other variables for which data were sought (e.g. participant and intervention characteristics, funding sources). Describe any assumptions made about any missing or unclear information.	p.4, Data extraction and management
Study risk of bias assessment	11	Specify the methods used to assess risk of bias in the included studies, including details of the tool(s) used, how many reviewers assessed each study and whether they worked independently, and if applicable, details of automation tools used in the process.	p.4, Quality appraisal
Effect measures	12	Specify for each outcome the effect measure(s) (e.g. risk ratio, mean difference) used in the synthesis or presentation of results.	Not applicable
Synthesis methods	13a	Describe the processes used to decide which studies were eligible for each synthesis (e.g. tabulating the study intervention characteristics and comparing against the planned groups for each synthesis (item #5)).	See Table 1
	13b	Describe any methods required to prepare the data for presentation or synthesis, such as handling of missing summary statistics, or data conversions.	p.4, Data analysis and presentation
	13c	Describe any methods used to tabulate or visually display results of individual studies and syntheses.	p.4, Data analysis and presentation
	13d	Describe any methods used to synthesize results and provide a rationale for the choice(s). If meta-analysis was performed, describe the model(s), method(s)	p.4, Data analysis

Section and Topic	Item #	Checklist item	Location where item is reported
		to identify the presence and extent of statistical heterogeneity, and software package(s) used.	and presentation
	13e	Describe any methods used to explore possible causes of heterogeneity among study results (e.g. subgroup analysis, meta-regression).	Not applicable
	13f	Describe any sensitivity analyses conducted to assess robustness of the synthesized results.	Not applicable
Reporting bias assessment	14	Describe any methods used to assess risk of bias due to missing results in a synthesis (arising from reporting biases).	Not applicable
Certainty assessment	15	Describe any methods used to assess certainty (or confidence) in the body of evidence for an outcome.	Not applicable
**RESULTS**			
Study selection	16a	Describe the results of the search and selection process, from the number of records identified in the search to the number of studies included in the review, ideally using a flow diagram.	p.5, Results
	16b	Cite studies that might appear to meet the inclusion criteria, but which were excluded, and explain why they were excluded.	Not applicable
Study characteristics	17	Cite each included study and present its characteristics.	See Table 1
Risk of bias in studies	18	Present assessments of risk of bias for each included study.	Supplement Table S4a-f
Results of individual studies	19	For all outcomes, present, for each study: (a) summary statistics for each group (where appropriate) and (b) an effect estimate and its precision (e.g. confidence/credible interval), ideally using structured tables or plots.	Not applicable
Results of syntheses	20a	For each synthesis, briefly summarise the characteristics and risk of bias among contributing studies.	p.5, Study characteristics
	20b	Present results of all statistical syntheses conducted. If meta-analysis was done, present for each the summary estimate and its precision (e.g. confidence/credible interval) and measures of statistical heterogeneity. If comparing groups, describe the direction of the effect.	See Figure 2 and Figure 3
	20c	Present results of all investigations of possible causes of heterogeneity among study results.	Not applicable
	20d	Present results of all sensitivity analyses conducted to assess the robustness of the synthesized results.	Not applicable
Reporting biases	21	Present assessments of risk of bias due to missing results (arising from reporting biases) for each synthesis assessed.	Not applicable
Certainty of evidence	22	Present assessments of certainty (or confidence) in the body of evidence for each outcome assessed.	Not applicable
**DISCUSSION**			
Discussion	23a	Provide a general interpretation of the results in the context of other evidence.	p.8f, Discussion,
	23b	Discuss any limitations of the evidence included in the review.	p.10, Challenges and limitations
	23c	Discuss any limitations of the review processes used.	p.10, Challenges and limitations
	23d	Discuss implications of the results for practice, policy, and future research.	p.10f, Conclusion
**OTHER INFORMATION**			
Registration and	24a	Provide registration information for the review, including register name and registration number, or state that the review was not registered.	p.3, Methods

Section and Topic	Item #	Checklist item	Location where item is reported
protocol	24b	Indicate where the review protocol can be accessed, or state that a protocol was not prepared.	p.3, Methods
	24c	Describe and explain any amendments to information provided at registration or in the protocol.	
Support	25	Describe sources of financial or non-financial support for the review, and the role of the funders or sponsors in the review.	To anonymise excluded from the manuscript
Competing interests	26	Declare any competing interests of review authors.	To anonymise excluded from the manuscript
Availability of data, code and other materials	27	Report which of the following are publicly available and where they can be found: template data collection forms; data extracted from included studies; data used for all analyses; analytic code; any other materials used in the review.	To anonymise excluded from the manuscript
